# Suppression of Antitumor Immune Responses by Human Papillomavirus through Epigenetic Downregulation of CXCL14

**DOI:** 10.1128/mBio.00270-16

**Published:** 2016-05-03

**Authors:** Louis Cicchini, Joseph A. Westrich, Tao Xu, Daniel W. Vermeer, Jennifer N. Berger, Eric T. Clambey, Denis Lee, John I. Song, Paul F. Lambert, Robert O. Greer, John H. Lee, Dohun Pyeon

**Affiliations:** a Department of Immunology and Microbiology, University of Colorado School of Medicine, Aurora, Colorado, USA; b Cancer Biology Research Center, Sanford Research, Sioux Falls, South Dakota, USA; c Department of Anesthesiology, University of Colorado School of Medicine, Aurora, Colorado, USA; d McArdle Laboratory for Cancer Research, University of Wisconsin—Madison, Madison, Wisconsin, USA; e Department of Otolaryngology, University of Colorado School of Medicine, Aurora, Colorado, USA; f Departments of Pathology and Dermatology and Division of Oral and Maxillofacial Pathology, University of Colorado School of Medicine, Aurora, Colorado, USA; g Department of Medicine, University of Colorado School of Medicine, Aurora, Colorado, USA

## Abstract

High-risk human papillomaviruses (HPVs) are causally associated with multiple human cancers. Previous studies have shown that the HPV oncoprotein E7 induces immune suppression; however, the underlying mechanisms remain unknown. To understand the mechanisms by which HPV deregulates host immune responses in the tumor microenvironment, we analyzed gene expression changes of all known chemokines and their receptors using our global gene expression data sets from human HPV-positive and -negative head/neck cancer and cervical tissue specimens in different disease stages. We report that, while many proinflammatory chemokines increase expression throughout cancer progression, *CXCL14* is dramatically downregulated in HPV-positive cancers. HPV suppression of *CXCL14* is dependent on E7 and associated with DNA hypermethylation in the *CXCL14* promoter. Using *in vivo* mouse models, we revealed that restoration of *Cxcl14* expression in HPV-positive mouse oropharyngeal carcinoma cells clears tumors in immunocompetent syngeneic mice, but not in *Rag1*-deficient mice. Further, *Cxcl14* reexpression significantly increases natural killer (NK), CD4^+^ T, and CD8^+^ T cell infiltration into the tumor-draining lymph nodes *in vivo*. *In vitro* transwell migration assays show that *Cxcl14* reexpression induces chemotaxis of NK, CD4^+^ T, and CD8^+^ T cells. These results suggest that *CXCL14* downregulation by HPV plays an important role in suppression of antitumor immune responses. Our findings provide a new mechanistic understanding of virus-induced immune evasion that contributes to cancer progression.

## IMPORTANCE

Human papillomaviruses (HPVs) are causally associated with more than 5% of all human cancers. During decades of cancer progression, HPV persists, evading host surveillance. However, little is known about the immune evasion mechanisms driven by HPV. Here we report that the chemokine *CXCL14* is significantly downregulated in HPV-positive head/neck and cervical cancers. Using patient tissue specimens and cultured keratinocytes, we found that *CXCL14* downregulation is linked to *CXCL14* promoter hypermethylation induced by the HPV oncoprotein E7. Restoration of Cxcl14 expression in HPV-positive cancer cells clears tumors in immunocompetent syngeneic mice, but not in immunodeficient mice. Mice with *Cxcl14* reexpression show dramatically increased natural killer and T cells in the tumor-draining lymph nodes. These results suggest that epigenetic downregulation of *CXCL14* by HPV plays an important role in suppressing antitumor immune responses. Our findings may offer novel insights to develop preventive and therapeutic tools for restoring antitumor immune responses in HPV-infected individuals.

## INTRODUCTION

Human papillomaviruses (HPVs) are causally associated with multiple human cancers, including cervical cancer (CxCa) and head and neck cancer (HNC) and result in about half a million deaths worldwide each year (1). Persistent infection of HPV is required for HPV-associated cancer development, and therefore, HPV must evade host immune surveillance (2). To evade host immune surveillance, HPV creates a local immune suppressive environment by inducing chemokine expression and diminishing the cytotoxic T cell response (2, 3). However, little is known about the mechanisms of disease progression driven by HPV-induced immune suppression.

To better understand the roles of host immunity in HPV-associated cancer progression, we analyzed the levels of expression of all known chemokines and chemokine receptors using our global gene expression data sets of CxCa progression (4) and HPV-positive and -negative HNCs (5). Deregulated chemokine networks in the tumor microenvironment (TME) alter immune cell infiltration, angiogenesis, and tumor cell growth, survival, and migration, leading to cancer progression (6). Recent laboratory studies and clinical trials have shown that restoring antitumor immune responses may be a promising therapeutic strategy to treat several cancers including HNCs (7–9). While initial studies have begun to explore relations between HPV infection and chemokine regulation, little is known about chemokine expression patterns altered by HPV during cancer progression. Here we show that, while expression of many proinflammatory chemokines is increased, *CXCL14* expression is significantly decreased in HPV-associated cancer progression.

CXCL14 (chemokine [C-X-C motif] ligand 14) is a chemokine distantly related to other CXC chemokines, showing 30% identity with CXCL2 and CXCL3 (10). CXCL14 functions as a potent angiogenesis inhibitor and a chemotactic factor for dendritic cells (DCs) and natural killer (NK) cells (11, 12). While normal human epithelial cells constitutively express *CXCL14*, its expression is frequently reduced in cervical, prostate, and oral cancers (13–15). Interestingly, restoration of *Cxcl14* expression recruits DCs into tumors *in vivo* and *in vitro* (15, 16) and induces tumor necrosis (17). Importantly, *Cxcl14* expression in HNC cells suppresses tumor growth from xenografts in athymic nude and SCID mice (18, 19). In addition, the rates of colorectal tumor formation and metastasis were significantly lower in *Cxcl14* transgenic mice than in isogenic wild-type mice (20). Previous studies have shown that CXCL14 inhibits signaling of proinflammatory chemokines interleukin 8 (IL-8) (11) and CXCL12 (21), which are known to promote cancer development and metastasis. Thus, CXCL14 has been suggested as a potential tumor suppressor having anti-inflammatory functions. *CXCL14* expression is epigenetically regulated by promoter hypermethylation in colorectal cancer cells (16). In the current investigation, we show that the *CXCL14* promoter is highly methylated and its expression is downregulated in HPV-positive tissues and cells in an E7-dependent manner. Importantly, restoration of murine *Cxcl14* expression in HPV-positive mouse oropharyngeal epithelial (MOE) cells increases NK, CD4^+^ T, and CD8^+^ T cell infiltration into the tumor-draining lymph nodes (TDLNs) and results in significant clearance of implanted HPV-positive HNC cells in immunocompetent syngeneic mice.

## RESULTS

### Proinflammatory chemokines are upregulated during CxCa progression.

To understand the mechanisms by which HPV deregulates host immune responses in the TME, we analyzed gene expression changes of known chemokines and their receptors in tissue epithelium during CxCa progression using our global gene expression data from human cervical tissue specimens of normal, cervical intraepithelial neoplasia grade 1 or 2 (CIN1/2), CIN3, or tumor tissues (GEO accession no. GSE63514) (4). The results showed that 14 chemokines and chemokine receptors increased at least threefold in cancer progression (see Table S1 in the supplemental material). Expression of *IL-8*, *CXCL9*, *CXCL11*, *CCL3*, and *CCL19* mRNAs was progressively increased throughout disease progression (see Fig. S1A in the supplemental material). In contrast, expression of *CXCL1*, *CXCL2*, *CXCL5*, *CXCL6*, and *CCL20* mRNA was significantly upregulated during the early transition from normal to CIN1/2 (Fig. S1B), while *CXCL13* and *CCL8* mRNA expression significantly increased only in the later transition to invasive tumors (Fig. S1C). Among chemokine receptors, *CXCR2* mRNA expression was decreased by 12-fold and *CXCR4* mRNA expression was upregulated nearly 7-fold throughout cancer progression (Fig. S1D). To identify HPV-specific chemokine deregulation, we analyzed our previously published gene expression data of HPV-positive and -negative HNCs (GEO accession no. GSE6791) (5). These results revealed that expression of *CXCL9*, *CXCL10*, *CXCL13*, and *CCL19* mRNAs as well as *CXCR4* mRNA was significantly upregulated in HPV-positive HNCs compared to HPV-negative HNCs (Fig. S2A to S2E), suggesting that HPV infection specifically changes chemokine expression. Unlike increased expression during cervical cancer progression, the expression level of *IL-8* mRNA was twofold lower in HPV-positive HNCs than in HPV-negative HNCs (Fig. S2F). Although HPV-positive cancers exhibit lower levels of *IL-8* expression compared to HPV-negative cancers, our previous study showed that *IL-8* expression was significantly increased in all HNCs compared to normal tissues (5). These results indicate that HPV-negative HNCs robustly upregulate *IL-8* and *CXCL1* expression more than HPV-positive HNCs by other mechanisms. To explore these changes of chemokine expression *in vitro*, we analyzed chemokine expression in cervical keratinocyte lines using reverse transcription quantitative PCR (RT-qPCR). We used W12E (derived from a low-grade precancerous cervical lesion with episomal human papillomavirus 16 [HPV16]), W12G (derived from a low-grade precancerous cervical lesion with integrated HPV16), and W12GPXY (transformed) cells which sequentially mimic CxCa progression (22). As expected, all W12 cell lines express high levels of the HPV16 early gene transcript (Fig. S2G). Expression levels of proinflammatory chemokines *IL-8*, *CXCL1*, *CXCL2*, *CXCL10*, and *CXCL11* were significantly increased in W12G and W12GPXY cells compared to a normal immortalized keratinocyte line (NIKS) (Fig. S2H to S2L). These results from tissue specimens and cultured keratinocytes suggest that several proinflammatory chemokines, which are recognized as major players in cancer development, are upregulated during HPV-associated cancer progression.

### *CXCL14* expression is downregulated in HPV-associated cancer progression.

**FIG 1:**
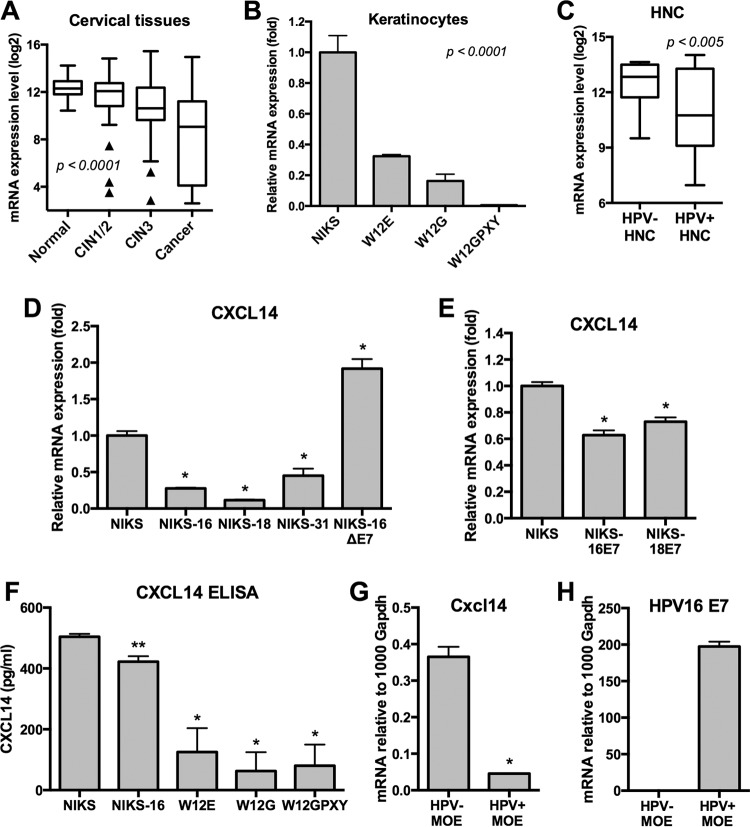
*CXCL14* expression is downregulated during HPV-associated cancer progression. (A and C) *CXCL14* mRNA expression levels were analyzed from global gene expression data sets of cervical tissue samples (A) and HNC tissue samples (C). (A) The 128 cervical tissue samples included samples in different disease stages (normal, *n* = 24; low-grade lesion, *n* = 36; high-grade lesion, *n* = 40; and cancer, *n* = 28) (4). (C) The 42 HNC tissue samples included 26 HPV-negative (HPV-) HNC and 16 HPV-positive (HPV+) (5) tissue samples. Normalized fluorescence intensities (log_2_) of gene expression from each group are shown in box-and-whisker plots with Tukey’s method for outliers (black triangles) noted as distinct data points. *P* values were determined by one-way ANOVA analysis (A) or Student’s *t* test (C). (B, D, and E) Total RNA was extracted from W12 (B) and NIKS (D and E) cell lines. The expression levels of *CXCL14* were measured by RT-qPCR. (F) Secreted CXCL14 was measured by ELISA using culture supernatant from NIKS, NIKS-16, W12E, W12G, and W12GPXY cells. (G and H) Total RNA was extracted from mouse oropharyngeal epithelial (MOE) cell lines, MOE/shPTPN13 (HPV negative) and MOE/E6E7 (HPV positive). The expression levels of murine *Cxcl14* mRNA (G) and HPV16 *E1^E4* mRNA transcript (H) were measured by RT-qPCR. HPV16 *E1^E4* and *CXCL14* mRNA copy numbers were calculated using serially diluted standard plasmids and normalized by human β-actin and murine *Gapdh* mRNA copy numbers. *P* values were calculated by Student’s *t* test. Values that are significantly different are indicated by asterisks as follows: *, *P* < 0.0001; **, *P* = 0.0002

While more than a dozen chemokines were highly upregulated, *CXCL14* was the only chemokine decreased more than threefold in CxCa progression (Fig. 1A). *CXCL14* mRNA expression was progressively decreased by about 21-fold from normal to cancer tissue. The downregulation of *CXCL14* was consistently observed in the W12 cell culture model (Fig. 1B). *CXCL14* expression levels showed a significant inverse correlation with the expression levels of *IL-8* and other proinflammatory chemokines in cervical tissue specimens and cultured keratinocytes (Fig. 1A and B; see Fig. S1A and Fig. S2H to S2L in the supplemental material). To determine whether *CXCL14* downregulation is unique to HPV-positive cancers, we compared *CXCL14* mRNA expression between HPV-positive and HPV-negative HNCs using the data sets from our previous global gene expression study (5). The results showed that *CXCL14* mRNA expression was significantly lower in HPV-positive HNC than in HPV-negative HNC (Fig. 1C). We also confirmed downregulation of *CXCL14* mRNA expression in HPV-positive HNC and CxCa compared to HPV-negative HNC using The Cancer Genome Atlas (TCGA) transcriptome sequencing (RNA-seq) data (23) (Fig. S3A). A previous study reported that *CXCL14* expression was significantly decreased in HNCs compared to normal tissue (13). Taken together, these results suggest that *CXCL14* is further downregulated in HPV-positive HNCs compared to HPV-negative HNCs and normal keratinocytes. To validate these observations using homogeneous keratinocyte culture models, we analyzed *CXCL14* mRNA expression in NIKS cell lines with and without high-risk HPV genomes. We found that each high-risk HPV (HPV16, HPV18, or HPV31) was sufficient to inhibit *CXCL14* expression (Fig. 1D). Interestingly, *CXCL14* expression was not downregulated in NIKS-16ΔE7 cells, which contain an E7-deficient HPV16 genome (24) (Fig. 1D). Further, *CXCL14* mRNA expression was modestly but significantly downregulated in NIKS cells expressing only the E7 oncoprotein from HPV16 or HPV18 (Fig. 1E). To detect secretion of the CXCL14 protein in cell culture supernatant, we performed an enzyme-linked immunosorbent assay (ELISA) using culture supernatant from NIKS and W12 cells. NIKS cells secreted a high level of CXCL14 protein, consistent with the previous study showing that normal keratinocytes constitutively express *CXCL14* (13). In contrast, CXCL14 levels secreted by NIKS-16 and W12 cells were significantly decreased, indicating that the *CXCL14* mRNA levels in NIKS and W12 cells correlate with CXCL14 secretion in cell culture supernatant (Fig. 1F). Taken together, these results suggest that the HPV oncoprotein E7 is sufficient to suppress *CXCL14* expression; however, long-term exposure is required for dramatic repression as seen in the W12GPXY cells and HPV-positive cancers.

Next, using HPV-positive and -negative MOE cells, we assessed the effect of the HPV16 oncoproteins E6 and E7 on murine *Cxcl14* expression. A protein sequence alignment demonstrated 98% homology between human CXCL14 and murine Cxcl14 within the C-X-C chemokine motif (data not shown). Two neutral amino acid substitutions are observed within the C-X-C motif: human CXCL14 I70 and V75, corresponding with murine Cxcl14 V58 and M63, respectively. We determined expression levels of *Cxcl14* mRNA in MOE cell lines, MOE/shPTPN13 (Ras transformed, HPV negative) and MOE/E6E7 (Ras transformed, expressing the HPV16 oncogenes E6 and E7) that form tumors in immunocompetent syngeneic C57BL/6 mice (25). Consistently, *Cxcl14* expression was also significantly downregulated in MOE/E6E7 cells compared to MOE/shPTPN13 cells (Fig. 1G and H). Taken together, our results suggest that *CXCL14* expression is specifically inhibited in HPV-positive cells, likely in an E7-dependent manner.

### *CXCL14* downregulation in HPV-positive keratinocytes is associated with promoter hypermethylation.

**FIG 2:**
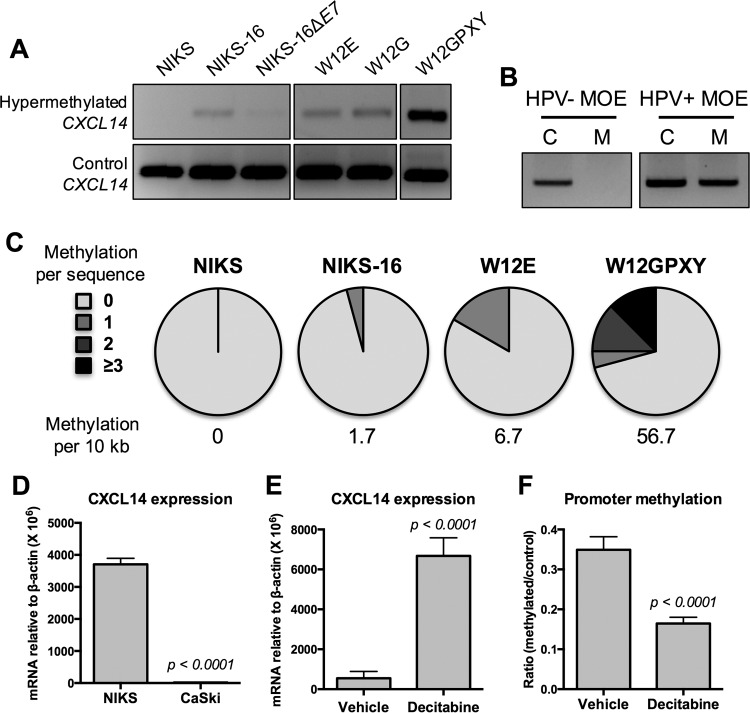
*CXCL14* downregulation in HPV-positive epithelial cells is associated with *CXCL14* promoter hypermethylation. (A and B) Genomic DNA was extracted from NIKS, NIKS-16, NIKS-16ΔE7, W12E, W12G, and W12GPXY cells (A) and MOE/shPTPN13 (HPV-negative) and MOE/E6E7 (HPV-positive) cells (B). MSP was performed using specific primers and analyzed in 1.2% agarose gel as described in Text S1 in the supplemental material. (B) MSP products of the control and hypermethylated *Cxcl14* promoter are shown in lanes C and M, respectively. (C) Bisulfite PCR products were cloned into the pGEM-T Easy vector and sequenced. (D) *CXCL14* expression was measured as described in the legend to Fig. 1. (E and F) CaSki cells were treated with 10 µM decitabine for 6 days or with a vehicle control (H_2_O) for 6 days. RT-qPCR (E) and qMSP (F) were performed using total RNA and genomic DNA, respectively. *CXCL14* mRNA copy numbers were normalized by β-actin mRNA (D and E). (F) Changes in *CXCL14* promoter methylation were calculated by using the 2^−ΔΔ*C*^_T_ method and shown as a fold ratio of methylated signal over total signal. *P* values were determined by Student’s *t* test.

Previous studies have shown that *CXCL14* expression is suppressed by DNA hypermethylation in the *CXCL14* promoter region (17). To determine whether HPV induces *CXCL14* promoter hypermethylation, we analyzed the methylation status of the *CXCL14* promoter in NIKS, NIKS-16, and W12 cell lines using methylation-specific PCR (MSP), as previously described (16). Interestingly, the *CXCL14* promoter region was hypermethylated in NIKS-16, W12E, W12G, and W12GPXY cells, but not in NIKS cells (Fig. 2A). Consistent with our results from cervical tissue specimens, the cervical keratinocyte lines W12E, W12G, and W12GPXY showed gradually increasing levels of *CXCL14* promoter hypermethylation during cancer progression (Fig. 2A). To determine whether the HPV oncoprotein E7 affects *CXCL14* promoter hypermethylation, we examined the methylation status of the *CXCL14* promoter in NIKS-16ΔE7 cells. Interestingly, *CXCL14* promoter hypermethylation was considerably lower in NIKS-16ΔE7 cells (Fig. 2A). These results indicate that the HPV16 E7 oncoprotein is necessary for HPV-induced *CXCL14* promoter hypermethylation. Next, we analyzed the DNA methylation status of the *CXCL14* promoter in HPV-positive versus -negative MOE cell lines. Consistent with our results from the keratinocyte culture models, the *CXCL14* promoter was hypermethylated in HPV-positive MOE cells, but not in HPV-negative MOE cells (Fig. 2B).

We further determined the methylation status of the CpG island within the promoter region of *CXCL14*, using bisulfite sequencing on genomic DNA from NIKS, NIKS-16, W12E, and W12GPXY cells. Promoter amplicons were cloned from genomic DNA, and 24 clones from each cell type were sequenced. Consistent with the MSP results above, there were no methylated cytidine residues detected in NIKS cells (Fig. 2C). Conversely, DNA methylation in the *CXCL14* promoter region appeared in NIKS-16 and W12E cells. A significantly higher frequency of *CXCL14* promoter methylation was found in the W12GPXY cell line, showing that ~25% of the *CXCL14* promoter clones contained multiple sites with DNA methylation (Fig. 2C). These results indicate that *CXCL14* promoter hypermethylation is induced by high-risk HPVs and accumulated over the course of cancer progression. This implies that other unknown factors in addition to E7 may be necessary for accumulation of *CXCL14* promoter hypermethylation in HPV-positive cells. To examine *CXCL14* promoter hypermethylation in HPV-positive cancer tissues, we analyzed *CXCL14* DNA methylation data from 279 HNC and 309 CxCa tissue samples obtained from the TCGA database (23). Consistent with our results from keratinocytes, *CXCL14* DNA methylation is significantly increased in HPV-positive HNC and CxCa compared to HPV-negative HNC (see Fig. S3B in the supplemental material). Interestingly, *CXCL14* downregulation is highly correlated with *CXCL14* DNA methylation in HPV-positive HNC and CxCa, but not in HPV-negative HNC (Fig. S3C to S3E). These results indicate that *CXCL14* mRNA expression is controlled by *CXCL14* promoter methylation in HPV-positive cancers. To verify *CXCL14* downregulation by promoter hypermethylation, we determined whether the methylation inhibitor decitabine (5-aza-2′-deoxycytidine) restores *CXCL14* mRNA expression (26). Unfortunately, decitabine was toxic to NIKS cells and ineffective in W12 cells to demethylate DNA at any concentration tested. We thus used an HPV16-positive CxCa cell line (CaSki), which also showed downregulated *CXCL14* expression (Fig. 2D). Decitabine treatment for 6 days significantly increased *CXCL14* expression in CaSki cells, corresponding with an approximately 50% decrease in *CXCL14* promoter methylation determined by quantitative MSP (qMSP) (Fig. 2E and F). These results suggest that reversing methylation at the *CXCL14* promoter, even partially, drastically increases *CXCL14* expression in HPV-positive cancer cells. Taken together, our results suggest that HPV downregulates *CXCL14* expression in HPV-positive cells by facilitating promoter hypermethylation.

### *CXCL14* expression hinders cell migration *in vitro*.

**FIG 3:**
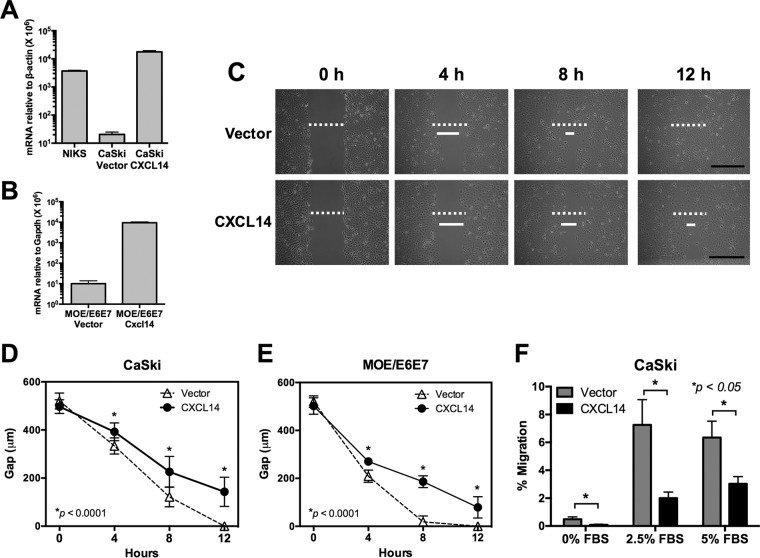
*CXCL14* expression hinders mobility of HPV-positive cancer cells. (A and B) CaSki and MOE/E6E7 cell lines reexpressing *CXCL14* were established using lentiviral transduction of the human *CXCL14* and murine *Cxcl14* genes, respectively, and validated by RT-qPCR. *CXCL14* and *Cxcl14* mRNA copy numbers were normalized by human β-actin or murine *Gapdh* mRNA, respectively. (C to E) *In vitro* scratch assay was performed with the established CaSki (C and D) and MOE/E6E7 (E) cells. Images were captured 0, 4, 8, and 12 h after the scratch or wound was generated, and the widths of the wound gaps were measured using NIH ImageJ software. Representative data from three replicates of each group are shown. The initial wound gaps (white dashed bar) and representative gaps at the indicated time points (solid white bar) are shown. Bars, 500 µm. (F) Transwell migration assays were performed on CaSki cells reexpressing *CXCL14* generated as described above for panel A. The percentage of cells that migrated through the permeable supports is shown, using 0%, 2.5%, and 5% FBS as a generic chemoattractant. *P* values were calculated by Student’s *t* test.

Previous studies have shown that CXCL14 interferes with IL-8 and CXCL12 signaling, which are important for tumor cell migration and invasion (11, 21). Consistently, *CXCL14* downregulation suppresses migration of colorectal and tongue cancer cell lines (27, 28). To determine the effects of *CXCL14* reexpression on HPV-positive cell migration, an *in vitro* scratch assay was performed. Unfortunately, NIKS and W12 cells differentiated and senesced when confluent, and therefore could not be used. Instead, we established CaSki and MOE/E6E7 cell lines reexpressing human *CXCL14* and murine *Cxcl14*, respectively, using lentiviral transduction (Fig. 3A and B). The expression level of *CXCL14* in CaSki cells was comparable to the level seen in NIKS (Fig. 3A). The results showed that *CXCL14* reexpression in both CaSki and MOE/E6E7 cells significantly delayed wound closure (Fig. 3C to E). While the gaps were filled within 8 h with control CaSki and MOE/E6E7 cells, both CaSki and MOE/E6E7 cells reexpressing *CXCL14* showed wide gaps of 50 to 200 µm 12 h after the scratch or wound was generated. To further corroborate these results, we performed a transwell migration assay using CaSki cells reexpressing *CXCL14* with fetal bovine serum (FBS) as a generic chemoattractant. The results showed that *CXCL14* expression significantly reduced CaSki cell migration compared to the vector-only control (Fig. 3F). *CXCL14* expression did not affect proliferation of CaSki and MOE/E6E7 cells (data not shown). Taken together, these results suggest that *CXCL14* downregulation in HPV-positive HNC and CxCa cells increases epithelial cell motility.

### Reexpression of *Cxcl14* clears HPV-positive tumors in immunocompetent mice, but not in *Rag1*-deficient mice.

**FIG 4:**
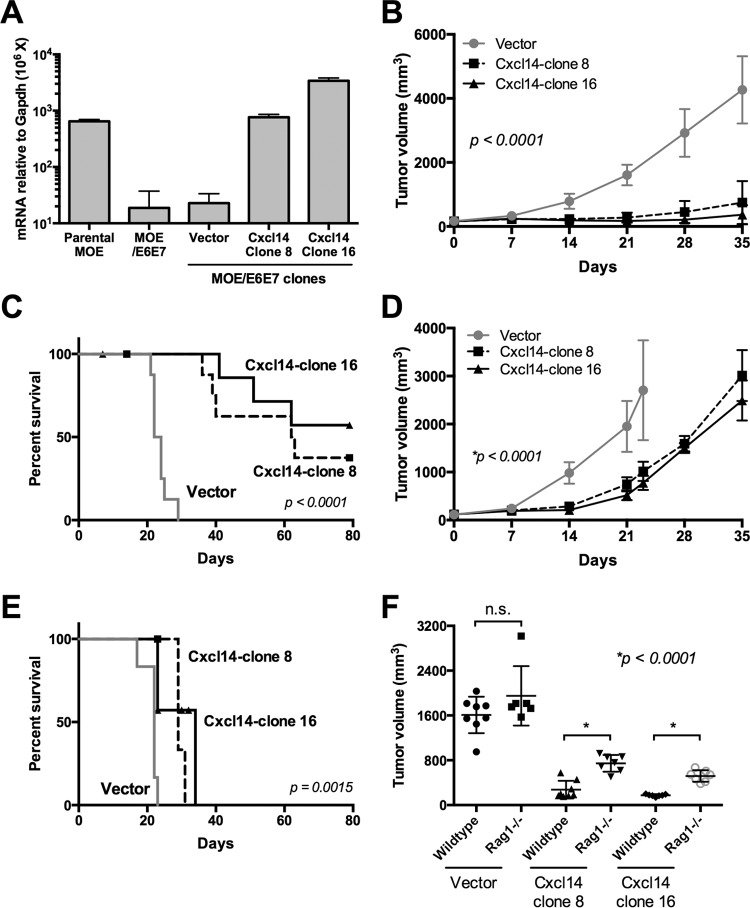
Restoration of *Cxcl14* expression clears HPV-positive tumors in immunocompetent mice, but not in *Rag1*-deficient mice. (A) MOE/E6E7 cell clones containing the *Cxcl14* gene or vector were established, and *Cxcl14* expression levels were determined by RT-qPCR. (B to F) Two MOE/E6E7 cell clones reexpressing *Cxcl14* (clones 8 and 16) and one vector containing MOE/E6E7 cell clone were injected into the rear right flank of wild-type C57BL/6 mice (B, C, and F) and *Rag1^−/−^* (D to F) C57BL/6 mice (*n* = 10 for each group of wild-type mice and *n* = 7 for each group of *Rag1^−/−^* mice). Tumor growth was determined every week by the following formula: volume = (width)^2^ × depth. (B to D) *P* value was determined by one-way ANOVA analysis (B and C) and Student’s *t* test (D). (E and F) Survival rates of wild-type and *Rag1^−/−^* mice were analyzed using a Kaplan-Meier estimator. The time to event was determined for each group (vector only, clone 8 reexpressing *Cxcl14* [*Cxcl14*-clone 8], clone 16 reexpressing *Cxcl14* [*Cxcl14*-clone 16]) with the event defined as a tumor burden larger than 2,500 mm^3^. Deaths not associated with tumor were censored. (F) Each symbol represents the value for an individual mouse. The mean (black bar) ± standard error of the mean (error bars) for each group of mice are shown. *P* values were determined by the log rank test (E and F). Values that were not significantly different (n.s.) are also shown.

To determine whether CXCL14 suppresses HPV-positive tumor growth *in vivo*, we established ~20 clones of MOE/E6E7 cells reexpressing various levels of murine *Cxcl14* using lentiviral transduction. Untransduced and vector-transduced MOE/E6E7 cells consistently showed a >30-fold decrease of *Cxcl14* mRNA expression compared to HPV-negative normal parental MOE cells (Fig. 4A). To understand the *in vivo* effects of *Cxcl14* reexpression, we chose to test two clones (clones 8 and 16) of our *Cxcl14*-reexpressing MOE/E6E7 cells that had physiological levels of *Cxcl14* mRNA expression comparable to parental MOE cells (Fig. 4A). *Cxcl14* reexpression did not affect proliferation of MOE/E6E7 cells (data not shown). Wild-type C57BL/6 mice were injected with 1 × 10^5^ MOE/E6E7 cells, from our established clones, in the rear right flank. Tumor growth was monitored by measuring tumor volume for up to 11 weeks. Strikingly, *Cxcl14* reexpression in MOE/E6E7 cells significantly suppressed tumor growth in wild-type C57BL/6 mice, while vector-transduced MOE/E6E7 cells rapidly formed tumors (Fig. 4B). All 10 mice transplanted with vector-transduced MOE/E6E7 cells succumbed to tumor burden within 5 weeks after injection (Fig. 4C). In contrast, 5 and 7 out of 10 mice transplanted with *Cxcl14*-reexpressing MOE/E6E7 clones 8 and 16, respectively, were tumor-free up to 11 weeks postinjection (Fig. 4C; see Fig. S4A to S4C in the supplemental material). To determine whether adaptive immune responses are involved in Cxcl14-mediated tumor suppression, we examined the tumor growth from these clones in *Rag1*-deficient C57BL/6 mice (*Rag1^−/−^*). Interestingly, tumor growth was moderately slowed by *Cxcl14* reexpression in *Rag1^−/−^* mice up to 14 days postinjection (Fig. 4D). However, all 14 *Rag1^−/−^* mice injected with clones 8 and 16 exhibited tumor growth and succumbed to tumor burden within 5 weeks postinjection (Fig. 4D and E and Fig. S4D to S4F). The results demonstrate no significant difference in tumor growth between wild-type and *Rag1^−/−^* mice transplanted with vector control MOE/E6E7 cells at 21 days postinjection (Fig. 4F). These results indicate that *Cxcl14* expression is critical to trigger an adaptive immune response to clear implanted cancer cells *in vivo*.

### Reexpression of *Cxcl14* increases natural killer, CD4^+^ T, and CD8^+^ T cells in tumor-draining lymph nodes *in vivo*.

**FIG 5:**
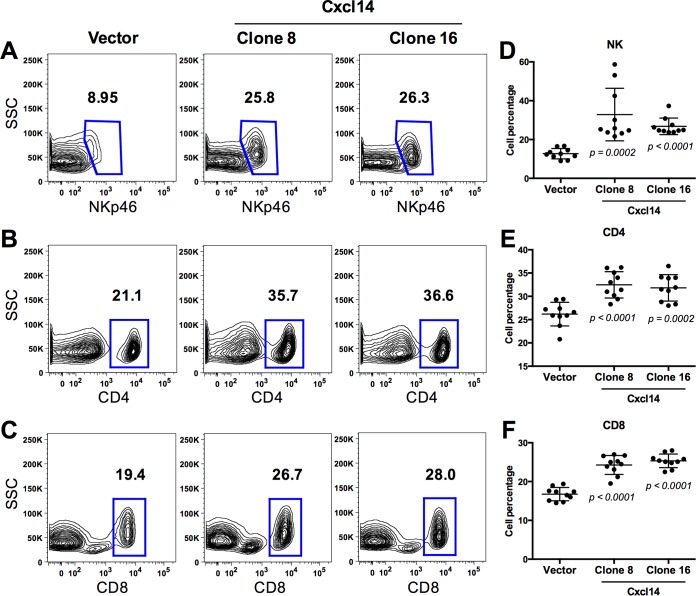
*Cxcl14* reexpression increases NK, CD4^+^ T, and CD8^+^ T cells in tumor-draining lymph nodes. MOE/E6E7 cells with *Cxcl14* (clones 8 and 16) or vector were injected into the rear right flank of C57BL/6 mice (*n* = 10 for each group of mice). Tumor-draining lymph nodes (TDLNs) were harvested from the mice 21 days postinjection. The percentage of immune cell populations defines the frequency of lymphocytes that were single cells and either NK (CD45^+^ NKp46^+^), CD4^+^ T (CD45^+^ CD4^+^), or CD8^+^ T (CD45^+^ CD8^+^) cells. Gating for flow cytometry was based on splenocyte populations and applied to TDLN samples as described in the legend to Fig. S5 in the supplemental material. (A to C) Representative flow cytometry diagrams and (D to F) quantification of the indicated immune cells in each mouse tested. *P* values were determined between vector and either clone 8 or clone 16 by Student’s *t* test. SSC, side scatter.

**FIG 6:**
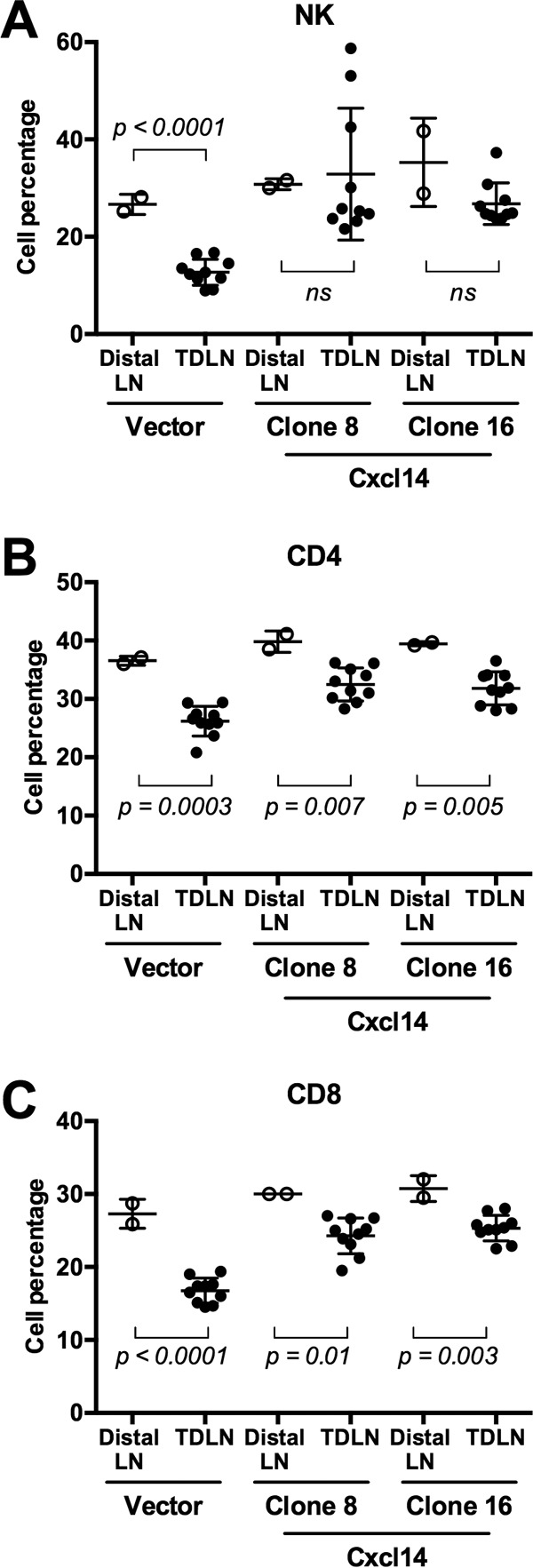
*Cxcl14* reexpression restores decreased populations of NK, CD4^+^ T and CD8^+^ T cells in TDLNs. Distal lymph nodes (distal LNs, open circles) and TDLNs (closed circles) were harvested from the mice injected with MOE/E6E7 cells reexpressing *Cxcl14* (clones 8 and 16) or containing vector only. The percentages of NK, CD4^+^ T, and CD8^+^ T cell populations were analyzed as described in the legend to Fig. 5. *P* values were determined between TDLN and distal LNs by Student’s *t* test. ns, not significant.

To characterize immune cell infiltration regulated by *Cxcl14* expression, we analyzed various immune cells in TDLNs and spleens harvested from the wild-type C57BL/6 mice at 21 days postinjection with vector-containing or *Cxcl14*-reexpressing MOE/E6E7 cells. Using flow cytometry, we assessed populations of hematopoietic cells (CD45^+^) including natural killer (NK) cells (NKp46^+^), CD4^+^ T cells, CD8^+^ T cells, antigen-presenting cells (major histocompatibility complex class II positive [MHCII^+^]), neutrophils (Gr1^high^), monocytes (Gr1^mid^), and macrophages (MHCII^+^ F4/80^+^). Our gating strategy for all interrogated cell types was based on cell populations detected in spleens and lymph nodes from C57BL/6 mice (see Fig. S5 in the supplemental material). Our data showed that percentages of NK, CD4^+^ T, and CD8^+^ T cells were highly increased in TDLNs of the mice transplanted with MOE/E6E7 cells reexpressing *Cxcl14* (Fig. 5). These results suggest that Cxcl14 increases infiltration of NK, CD4^+^ T, and CD8^+^ T cells into TDLNs, which may be critical for tumor clearance. This is consistent with our tumor growth results showing a moderate delay in tumor growth by *Cxcl14* reexpression in *Rag1^−/−^* mice, in which NK cell infiltration is increased in the absence of T cells (data not shown). These results suggest that NK cells alone may not be sufficient to clear HPV-associated tumors (Fig. 4D and E). In addition to increased NK, CD4^+^ T, and CD8^+^ T cell infiltration, monocytes were also modestly increased in TDLNs of the mice injected with MOE/E6E7 cells reexpressing *Cxcl14*. Conversely, *Cxcl14* reexpression did not change antigen-presenting cells, neutrophils, and macrophages in TDLNs (Fig. S6), and marginal or no changes of these immune cell populations were observed in spleens by *Cxcl14* reexpression (Fig. S7). These results indicate that Cxcl14 locally affects NK, CD4^+^ T, and CD8^+^ T cell infiltration near the TME. To determine any difference in local and systemic immune responses altered by Cxcl14, populations of NK, CD4^+^ T, and CD8^+^ T cells were compared between TDLNs and distal lymph nodes (LNs) in the same mice injected with MOE/E6E7 cells with or without *Cxcl14* reexpression. Interestingly, NK, CD4^+^ T, and CD8^+^ T cell populations were significantly decreased in TDLNs than in distal LNs in mice injected with control MOE/E6E7 cells (Fig. 6). In contrast, mice injected with MOE/E6E7 cells reexpressing *Cxcl14* showed significantly restored NK, CD4^+^ T, and CD8^+^ T cell populations in TDLNs comparable to distal LNs (Fig. 6). These results indicate that reexpression of *Cxcl14* reverses suppression of antitumor immune responses by locally recruiting NK, CD4^+^ T, and CD8^+^ T cells.

### Reexpression of *Cxcl14* induces chemotaxis of NK, CD4^+^ T, and CD8^+^ T cells *in vitro*.

**FIG 7:**
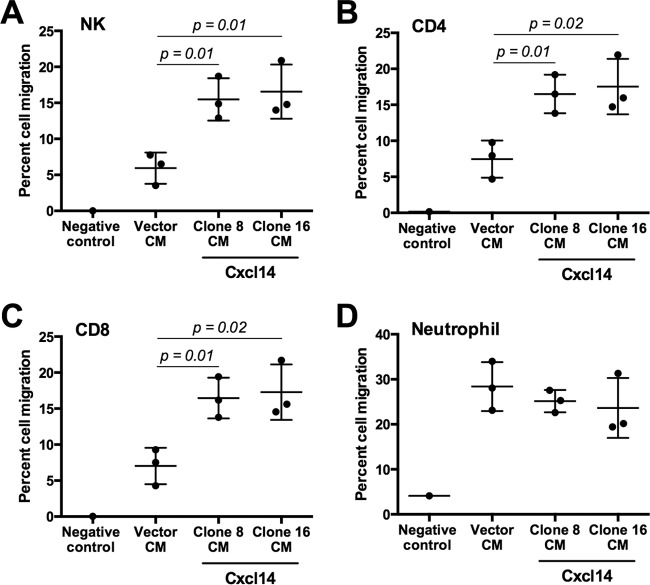
Cxcl14 reexpression induces chemotaxis of NK, CD4^+^ T, and CD8^+^ T cells. Conditioned media (CM) from the culture of MOE cells with *Cxcl14* (clones 8 and 16) or vector were added into the bottom chamber of a transwell and supplemented with IL-2. Splenocytes isolated from C57BL/6 mice were added to the top chamber. After 12-h incubation, migrated splenocytes to the bottom chamber were collected and analyzed by flow cytometry. The percentage of immune cell populations defines the frequency of immune cells that were single cells and either NK (CD45^+^ NKp46^+^) (A), CD4^+^ T (CD45^+^ CD4^+^) (B), CD8^+^ T (CD45^+^ CD8^+^) cells (C), or neutrophils (CD45^+^ Gr1^high^) (D). *P* values were determined between vector-containing and *Cxcl14*-reexpressing cells (clones 8 and 16) by Student’s *t* test.

To determine whether reexpression of *Cxcl14* in MOE/E6E7 cells induces chemotaxis of NK, CD4^+^ T, and CD8^+^ T cells, we performed an immune cell migration assay using the transwell system and splenocytes isolated from C57BL/6 mice. The results showed that conditioned medium from cultured MOE/E6E7 cells reexpressing *Cxcl14* (clones 8 and 16) significantly increased NK, CD4^+^ T, and CD8^+^ T cell chemotaxis, while conditioned medium from MOE/E6E7 cells containing vector only has little effect compared to the negative control (Fig. 7A to C). Consistent with the *in vivo* immune cell infiltration (see Fig. S6B in the supplemental material), neutrophil migration was not affected by *Cxcl14* reexpression (Fig. 7D). These results suggest that Cxcl14 plays an important role in recruitment of NK, CD4^+^ T, and CD8^+^ T cells, which may enhance antitumor immune responses.

## DISCUSSION

Like most cancers, HPV-associated cancer development requires decades to progress from HPV-infected cells to invasive disease. Recent cancer genomics studies of HNCs have reported that HPV-positive HNCs have far fewer oncogenic mutations (~5 per tumor) than HPV-negative HNCs do (>20 per tumor) (29). These findings indicate that viral factors replace oncogenic processes usually triggered by multiple somatic mutations in HPV-unrelated cancer progression. Other studies showed that continuous expression of the HPV oncogene E7 is required for cancer growth and maintenance *in vitro* and *in vivo* (30, 31), suggesting that HPV E7 has multiple functions in HPV-associated cancer progression. However, the mechanism by which HPV infection contributes to multiple steps of decades-long cancer progression is poorly understood.

Recent studies have shown that several proinflammatory chemokines such as IL-8, CXCL1, and CXCL12 drive cancer progression by facilitating tumor cell growth, survival, and migration as well as by inducing angiogenesis (32). In our study, expression of proinflammatory chemokines *IL-8*, *CXCL1*, *CXCL2*, and *CCL3* was upregulated in the early stages of cancer progression (see Fig. S1A and S1B in the supplemental material). These chemokines are also increased in HPV-negative HNCs but to higher levels than HPV-positive HNCs, suggesting that most HNCs might have increased levels of proinflammatory chemokine expression that is pivotal for tumor cell migration and angiogenesis (33).

Interestingly, *CXCL14* was significantly downregulated during CxCa progression and in HPV-positive HNCs compared to HPV-negative HNCs (Fig. 1A and C). Constitutively expressed in normal epithelial and neural tissue in mammals, CXCL14 is thought to be an important homeostatic chemokine (13, 34, 35). Additionally, by directly binding to IL-8, CXCL14 inhibits the ability of IL-8 to recruit endothelial cells and promote angiogenesis (11), which is known to be essential for cancer progression. While specific receptors of CXCL14 have not been identified, a recent study showed that CXCL14 binds to CXCR4 (chemokine [C-X-C motif] receptor 4) as a decoy ligand, inhibiting CXCL12 signal transduction through CXCR4 (21), an important signaling pathway for cell growth, angiogenesis, and metastasis in many cancers. *CXCL14* expression is frequently downregulated in cervical, prostate, colorectal, lung, and oral cancers (13–16, 18, 36, 37). Overexpression of *CXCL14* has shown antitumor effects by suppressing tumor growth and cancer cell migration in breast, oral, lung, and liver cancers (17–19, 38, 39). Consistently, our results here show that *CXCL14* reexpression in HPV-positive cells significantly suppresses tumor growth *in vivo* (Fig. 4).

Additionally, the HPV oncoprotein E7 induces *CXCL14* promoter hypermethylation and significantly downregulates *CXCL14* expression (Fig. 1 and 2). A previous study found that HPV16 E7 activates the methyltransferase activity of DNMT1 (DNA methyltransferase 1) (40). Our preliminary study also showed upregulation of *DNMT1* expression in HPV-positive cancers and keratinocytes (data not shown). These observations suggest that *CXCL14* promoter methylation may be mediated through an E7 interaction with DNMT1. *CXCL14* expression in HPV-positive CaSki cells was significantly increased following treatment with decitabine, a FDA-approved DNMT inhibitor (41) (Fig. 2E). Previous studies have shown that DNA hypermethylation is associated with suppression of various immune factors including downregulation of cancer testis antigen, MHC molecule, and chemokine expression (42). Consistently, inhibition of DNA methylation by decitabine increases expression of cancer testis antigens and MHC molecules and enhances cytotoxic NK and T cell antitumor activity (43–45). Decitabine treatment also activates expression of several different chemokines in a murine ovarian cancer model (46). Similarly, a recent study showed that decitabine treatment enhanced antitumor immune responses by increasing *CXCL9* and *CXCL10* expression and effector T cell infiltration (47). Thus, reversing the promoter hypermethylation of *CXCL14* could be a feasible approach for restoring antitumor immune responses to treat HPV-positive cancers.

In our current study, we assessed the potential for CXCL14 to alter immune cell infiltration in TDLNs. We showed that restoration of *Cxcl14* expression increases the percentages of NK, CD4^+^ T, and CD8^+^ T cell populations in TDLNs (Fig. 5). Because tumor growth is only partially suppressed by *Cxcl14* reexpression in *Rag1^−/−^* mice, our results indicate that both innate and adaptive immune responses play important roles in the antitumor functions of CXCL14. A marked reduction in NK cell activity in uterine walls was consistently observed in Cxcl14^−/−^ mice compared to Cxcl14^+/−^ mice (37). In addition, NK cell depletion increases the risk of colorectal cancer in *Cxcl14* transgenic mice (20). On the other hand, the effects of CXCL14 on T cells are completely unknown. Both NK and CD8^+^ T cells are well-known as effector killer cells capable of eliminating virus-infected cells as well as cancer cells (48–51). NK cell activation induces CD8^+^ T cell responses through priming DCs, suggesting that NK cells may be the link between innate and adaptive immunity to induce antiviral and antitumor CD8^+^ T cell responses (51, 52). Thus, our findings suggest that CXCL14, secreted by epithelial cells, might be one of the key regulators for NK, CD4^+^ T, and CD8^+^ T cells to drive tumor clearance during HPV-associated cancer progression.

In conclusion, our study suggests that CXCL14 plays an important role in antitumor immune responses to clear HPV-positive HNC. CXCL14 is a small, secreted protein that can be used as a therapeutic agent. Additionally, identification of the native CXCL14 receptor(s) would provide druggable targets to enhance CXCL14 functions. Thus, further studies of the effects of CXCL14 on NK and T cells may provide a novel means of anticancer immunotherapy to treat HNCs.

## MATERIALS AND METHODS

### Cell lines.

Human keratinocytes NIKS cells were obtained from Allen-Hoffmann (53). NIKS-16, NIKS-18, NIKS-31 (54), W12E (derived from a low-grade precancerous cervical lesion with episomal HPV16) (55), and W12G (derived from a low-grade precancerous cervical lesion with integrated HPV16) (56) cells were established in the Paul Lambert laboratory and grown with NIH 3T3 feeder mouse fibroblasts as described previously (5). Transformed W12GPXY cells derived from W12G cells were obtained from Sheila Graham (University of Glasgow) in 2011. NIKS and NIKS derivatives were generated in 1999 and maintained under passage 50 as previously described (53, 54). NIKS and W12 cells were validated by morphology, HPV early gene expression, and feeder cell dependency. CaSki cells were obtained from the American Type Culture Collection (ATCC) in 1987 (57) and validated by HPV early gene expression. The mouse oropharyngeal epithelial (MOE) cell lines MOE/shPTPN13 (transformed with Ras and short hairpin RNA [shRNA] against Ptpn13 [protein tyrosine phosphatase, nonreceptor type 13]) and MOE/E6E7 (transformed with Ras and HPV16 E6/E7) were generated by John Lee in 2009 (25) and validated by assessing cytokeratin expression, the presence of the E6 and E7 expression vectors which confer resistance to puromycin, and activation of the mitogen-activated protein kinase (MAPK) pathway, a hallmark of E6 expression. All cell lines were cultured according to the suppliers’ recommendations.

### Reverse transcription-qPCR (RT-qPCR).

Total RNA was extracted from keratinocytes (Qiagen), and first strand cDNA was reverse transcribed using SuperScript II reverse transcriptase (Life Sciences). Real-time PCR was performed using SYBR green (Roche). Primer sequences appear in Table S2 in the supplemental material. Data were normalized by the level of β-actin or *Gapdh* mRNA.

### Bisulfite modification, methylation-specific PCR (MSP), and bisulfate sequencing.

Genomic DNA was extracted from keratinocytes (Qiagen) and bisulfite converted using EZ DNA methylation kit (Zymo Research). Bisulfite sequencing products were cloned into pGEM-T Easy vector (Promega) and sequenced. Quantitative MSP (qMSP) was performed with bisulfite-converted genomic DNA using SYBR green (Roche). Relative DNA methylation was calculated using the Δ*C_T_* equation using methylated DNA as the target.

### Cell migration assays.

Confluent CaSki and MOE/E6E7 monolayers were scratched, and the width of the gap was measured every 4 h. The transwell migration assay was performed using 1 × 10^5^ cells per well of an 8-µm 24-well transwell permeable support and incubated overnight using FBS as a chemoattractant. Spleens from C57BL/6 mice injected with MOE/E6E7 cells were harvested at 21 days postinjection and mechanically disrupted through a 100-µm filter. Red blood cells (RBC) were cleared by RBC lysis buffer (Sigma), and the remaining splenocytes were allowed to rest at 37°C in RPMI 1640 medium containing 10% FBS and 10 ng/ml of mouse recombinant IL-2 (mrIL-2) (eBioscience) for 3 h. Conditioned media (CM) from the culture of MOE cells reexpressing *Cxcl14* (clones 8 and 16) or containing vector were added into the bottom chamber of a transwell (3-μm pore size; Costar). Isolated splenocytes (2 × 10^6^ cells/ml) were resuspended in RPMI 1640 medium supplemented with mrIL-2 and added to the top chamber of the transwell. Splenocytes in RPMI 1640 medium without mrIL-2 were used as a negative control. After 12-h incubation at 37°C, splenocytes were harvested from the top and bottom chambers, stained with trypan blue, and counted using a hemocytometer. Cell populations were analyzed using flow cytometry as described below in “Antibodies and flow cytometry.” Total cell populations were determined by applying the cell counts to the cell population percentages. The migration index for each cell type was calculated by the following equation; percent cell migration = migrated cell number (bottom chamber)/total cell number (top chamber + bottom chamber).

### Mice and treatment.

Four- to 6-week-old, 20- to 25-g male C57BL/6J wild-type or *Rag1^−/−^* mice (The Jackson Laboratory) were maintained in accordance with the USDA guidelines. Tumors were initiated by injection of engineered MOE/E6E7 cells (1 × 10^5^) subcutaneously into the rear right flank of mice (*n* = 10 per group). Tumor growth was measured weekly using previously established techniques (58). Tumor volume was calculated using the following equation: volume = (width)^2^ × depth. Animals were euthanized when the tumor size was greater than 1.5 cm in any dimension. Conversely, mice were considered tumor free when no measurable tumor was detected for a period of 11 weeks. Survival graphs were calculated by standardizing for a tumor volume of 2,500 mm^3^.

### Antibodies and flow cytometry.

For each experimental group, TDLNs and spleens were harvested from wild-type mice 21 days after injection. The following anti-mouse antibodies were purchased from eBioscience and used according to the manufacturer’s specifications: MHCII (fluorescein isothiocyanate [FITC] conjugate, clone M5/114.15.2), CD4 (eF450 conjugate, clone RM4-5), F4/80 (allophycocyanin [APC] conjugate, clone BM8), and Gr1 (AF700 conjugate, RB6-8C5). Anti-mouse CD45 (peridinin chlorophyll protein [PerCP] conjugate, clone 30-F11) and NKp46 (phycoerythrin-Cy7 [PECy7] conjugate, clone PC61.5) were purchased from BioLegend. All tissue samples were passed through a 100-µm cell strainer (Corning Life Sciences), and spleens were incubated in red blood cell lysing buffer Hybri-Max (Sigma-Aldrich) for 3 min at room temperature. The isolated cells were incubated with a panel of antibodies conjugated with unique fluorophores for 1 h at room temperature and washed with phosphate-buffered saline (PBS). Samples were passed through a 35-µm cell strainer (Corning Life Sciences) immediately before analysis on an LSRII flow cytometer (Becton Dickinson) using FACSDiva collection software. All cells were assessed for viability by staining with LIVE/DEAD fixable aqua dead cell stain (Life Technologies). Analysis was performed using FlowJo software, and the gating strategy is described in the legend to Fig. S5 in the supplemental material.

### Statistical analysis.

Student’s *t* test and one-way analysis of variance (ANOVA) were used to calculate significance for comparison of two matched groups and three or more unmatched groups, respectively. The correlation coefficient (*R*^2^) was determined by linear regression using Prism 6 (GraphPad). Results were considered statistically significant at a *P* value of less than 0.05. The distributions of time to event outcomes (e.g., survival time) were summarized with Kaplan-Meier curves and compared across groups using the log rank test with α = 0.01.

## SUPPLEMENTAL MATERIAL

Text S1  Supplemental methods and materials, including ethics statement, reverse transcription-qPCR, bisulfite modification, methylation-specific PCR, bisulfate sequencing, expression vectors, and enzyme-linked immunosorbent assay. Download Text S1, PDF file, 0.1 MB

Figure S1  Chemokine expression is deregulated in HPV-associated cancer progression. Chemokines and chemokine receptors with significant changes of expression in CxCa progression are shown in the different panels. (A) *IL-8*, *CXCL9*, *CXCL11*, *CCL3*, and *CCL19*; (B) *CXCL1*, *CXCL2*, *CXCL5*, *CXCL6*, and *CCL20*; (C) *CXCL13* and *CCL8*; and (D) *CXCR2* and *CXCR4*. The gene expression data were analyzed from a global gene expression study of 128 cervical tissue samples in different disease stages: normal (*n* = 24); low-grade lesion (*n* = 36); high-grade lesion (*n* = 40); and cancer (*n* = 28) (4). Normalized fluorescence intensities (log_2_) of gene expression from each group are shown in box-and-whisker plots with Tukey’s method for outliers (black triangles) noted as distinct data points. *P* values were calculated between each transition (normal to CIN1/2, CIN1/2 to CIN3, and CIN3 to cancer) by Student’s *t* test. *, *P* < 0.05. Download Figure S1, JPG file, 0.5 MB

Figure S2  Proinflammatory chemokines are upregulated in HPV-positive HNCs and keratinocytes. (A to F) Gene expression levels of chemokines and chemokine receptors were analyzed with HPV-positive (*n* = 16) and HPV-negative (*n* = 26) HNCs from our previous global gene expression study (5) as described in the legends to Fig. 1 and Fig. S1 in the supplemental material. Normalized fluorescence intensities (log_2_) of gene expression from each group are shown in box-and-whisker plots with Tukey’s method for outliers (black circle) noted as distinct data points. The *P* values shown on each panel were calculated for HPV-negative and HPV-positive HNCs by Student’s *t* test. (G to L) Total RNA was extracted from NIKS, W12E, W12G, and W12GPXY keratinocyte lines. (G) HPV16 early gene transcript E1^E4 was measured by RT-qPCR, as previously described (59). (H to L) mRNA expression of *IL-8*, *CXCL1*, *CXCL2*, *CXCL10*, and *CXCL11* were measured by RT-qPCR using specific primers (see Table S2 in the supplemental material), and normalized by β-actin mRNA. Data are shown as fold changes (± SD) to the mRNA level in NIKS cells. *P* values were determined by Student’s *t* test. *, *P* < 0.05; **, *P* < 0.001; ***, *P* < 0.0001. Download Figure S2, JPG file, 0.7 MB

Figure S3  *CXCL14* downregulation correlates with increased *CXCL14* promoter methylation in HPV-positive HNC and CxCa. The TCGA data sets of *CXCL14* RNA-seq RSEM (RNA-seq by expectation maximization) counts (mRNA expression) and beta values (DNA methylation) were obtained from cBioPortal (cbioportal.org): HPV-negative HNC, *n* = 243; HPV-positive HNC, *n* = 36 (23); CxCa, *n* = 309 (NCI, TCGA, Provisional). Normalized RSEM counts (A) and beta values (B) are shown in box-and-whisker plots with Tukey’s method for outliers (black triangles) noted as distinct data points. *P* values were determined by Student’s *t* test. *n.s.*, not significant. Correlations between *CXCL14* mRNA expression and DNA methylation were analyzed within HPV-positive (HPV+) HNC (C), HPV-negative (HPV−) HNC (D), and CxCa (E). The correlation coefficient (*R*^2^) was determined by linear regression using Prism software. Download Figure S3, JPG file, 0.6 MB

Figure S4  Restoration of *Cxcl14* expression suppresses tumor growth *in vivo*. MOE/E6E7 cell clones reexpressing *Cxcl14* (clones 8 and 16) and a vector containing MOE/E6E7 cell clone were injected into the rear right flank of wild-type C57BL/6 (A to C) and *Rag1^−/−^* (D to F) mice (*n* = 10 for each group of wild-type mice; *n* = 7 for each group of *Rag1^−/−^* mice). Tumor growth was determined every week by the following formula: volume = (width)^2^ × depth. Tumor growth curves of each mouse are shown. Download Figure S4, JPG file, 0.3 MB

Figure S5  Gating strategy for flow cytometry. The whole spleen from a C57BL/6 mouse was homogenized and stained with a panel of antibodies conjugated to unique fluorophores. Single-stain and no-stain controls were used for fluorescence compensation. A generous large cell gate (forward scatter versus side scatter area), single cell gate (side scatter area versus side scatter width), and CD45^+^ gate (side scatter area versus CD45) were applied as parental gates before determining antigen-presenting cell (side scatter area versus MHCII), neutrophil (side scatter high, Gr1^high^), monocyte (side scatter low, Gr1^mid^), and macrophage (MHCII^+^ F4/80^+^) populations. A small cell lymphocyte gate (side scatter area versus forward scatter) and single cell gate (side scatter area versus side scatter width) were applied as parental gates to determine NK cell (CD45^+^ NKp46^+^), CD4^+^ T cell (CD45^+^ CD4^+^), and CD8^+^ T cell (CD45^+^ CD8^+^) populations. A representative example of the overall gating strategy is shown and was applied to TDLNs, distal lymph nodes, and spleens harvested from C57BL/6 mice injected with MOE/E6E7 cells with Cxcl14 or vector. Download Figure S5, JPG file, 0.6 MB

Figure S6  Changes of immune cell populations in TDLNs by *Cxcl14* reexpression. MOE/E6E7 cells with *Cxcl14* (clones 8 and 16) or the vector were injected into the right flank of C57BL/6 mice (*n* = 10 for each group). TDLNs were harvested 21 days postinjection. The percentages of antigen-presenting cell (A), neutrophil (B), monocyte (C), and macrophage (D) populations were determined by flow cytometry using specific antibodies as described in Materials and Methods. *P* values were determined between vector alone and clone 8 or clone 16 by Student’s *t* test. Download Figure S6, JPG file, 0.3 MB

Figure S7  Changes of immune cell populations in spleens by *Cxcl14* reexpression. MOE/E6E7 cells with *Cxcl14* (clones 8 and 16) or the vector were injected into the right flank of C57BL/6 mice (*n* = 10 for each group). Spleens were harvested at 21 days postinjection. The percentages of NK cell (A), CD4^+^ T cell (B), CD8^+^ T cell (C), antigen-presenting cell (D), neutrophil (E), monocyte (F), and macrophage (G) populations were determined by flow cytometry using specific antibodies as described in Materials and Methods. *P* values were determined between vector alone and clone 8 or clone 16 by Student’s *t* test. Download Figure S7, JPG file, 0.4 MB

Table S1  Chemokine expression profiles of tissue specimens from cervix and head/neck. Table S1, PDF file, 0.1 MB

Table S2  Quantitative PCR primers. Table S2, PDF file, 0.05 MB
