# Supplementary material for: Suppression of Antitumor Immune Responses by Human Papillomavirus through Epigenetic Downregulation of CXCL14
Source: mBio. 2016 May 3;7(3):e00270-16. doi: 10.1128/mBio.00270-16 (PMC4959654; doi:10.1128/mBio.00270-16)
Supplement: Text S1 — Supplemental methods and materials, including ethics statement, reverse transcription-qPCR, bisulfite modification, methylation-specific PCR, bisulfate sequencing, expression vectors, and enzyme-linked immunosorbent assay. Download [file mbo002162801s1.pdf]

SUPPLEMENTAL METHODS

Ethics Statement

All animal experiments were performed in strict accordance with the Guidelines for the Care and Use of Laboratory Animals of the National Institutes of Health. The protocols were approved by the the Institutional Animal Care and Use Committees of University of Colorado Denver (Assurance Number: A3269-01) and Sanford Research (Assurance Number: A4568-01).

Reverse Transcription-qPCR (RT-qPCR)

Total RNA was extracted from keratinocytes using RNeasy mini kit (Qiagen) and in column DNA digest was performed using RNase-free DNase set (Qiagen) according to the manufacturer's instructions. First strand complementary DNA (cDNA) was reverse transcribed with SuperScript II Reverse Transcriptase (Life Sciences) and oligo-d$(T)_{16}$ (Integrated DNA Technologies) from total RNA. Real-time PCR was performed using the Bio-Rad CFT Connect Real-time System and FastStart Universal SYBR Green Master (Rox) (Roche Applied Science). Primer sequences appear in **Table S2**. Data were normalized by the level of ß-actin (*ACTB*) or *Gapdh* mRNA in each human or mouse sample, respectively.

Bisulfite Modification, Methylation-Specific PCR (MSP), and Bisulfate Sequencing

The methylation status of the *CXCL14* promoter in HPV-positive or -negative keratinocyte cells was analyzed by MSP. Genomic DNA was extracted from keratinocytes using DNeasy Blood & Tissue kit (Qiagen). 500 ng of the extracted genomic DNA was used for bisulfite treatment with the EZ DNA Methylation kit (Zymo Research) according to the manufacturer's instruction. MSP was performed using the following forward and reverse primers: 5'-GGTTGGGAAGGTTTTTTTT-3' and 5'-ACCCAACTCTACTCRACTTTCT-3'. MSP and bisulfite sequencing primers were used as previously described [16]. Bisulfite sequencing products were cloned into pGEM-T easy vector (Promega) and 24 clones for each cell type were sequenced with T7 primer (Beckman Coulter Genomics). qMSP was performed with bisulfite-converted genomic DNA using FastStart Universal SYBR Green Master (Rox) (Roche Diagnostics). Relative DNA methylation was calculated using the ΔCt equation using methylated

DNA as target, the non-specific control as reference.

Expression Vectors

The murine *Cxcl14* expression vector was generated using the following forward and reverse primers to amplify the *Cxcl14* sequence using mRNA extracted from MOE cells: 5'-ATTAGAATTCATGAGGCTCCTGGCGGCCGC-3' and 5'-ATCGGGATCCCTATTCTTCGTAGACCCTGC-3', respectively. The EcoRI and BamHI restriction sites (underlined) were used to clone the amplified Cxcl14 cDNA sequence into the pCDH-CMV-MCS-EF1-Puro lentiviral expression vector (CD510B-1, System Biosciences). The human CXCL14 lentiviral expression construct was purchased from the Functional Genomics Facility at the University of Colorado Boulder. Lentiviruses were propagated in 293FT cells according to the protocol supplied by the manufacturer (System Biosciences).

Enzyme-Linked Immunosorbent Assay (ELISA)

All ELISA reagents were purchased from R&D Systems. CXCL14 concentration was determined using DuoSet ELISA Development System for Human BRAK/CXCL14 according to the manufacturer's specifications. Absorbance was determined using the Bio-Tek Synergy HT plate reader and analyzed using Bio-Tek KC4 software. CXCL14 concentration was determined from a standard curve of recombinant human CXCL14 using the 4-parameter logistic regression model from ElisaAnalysis.com (LTG Ventures Pty Ltd).
