# Supplementary figures and images for: Suppression of Antitumor Immune Responses by Human Papillomavirus through Epigenetic Downregulation of CXCL14
Source: mBio. 2016 May 3;7(3):e00270-16. doi: 10.1128/mBio.00270-16 (PMC4959654; doi:10.1128/mBio.00270-16)

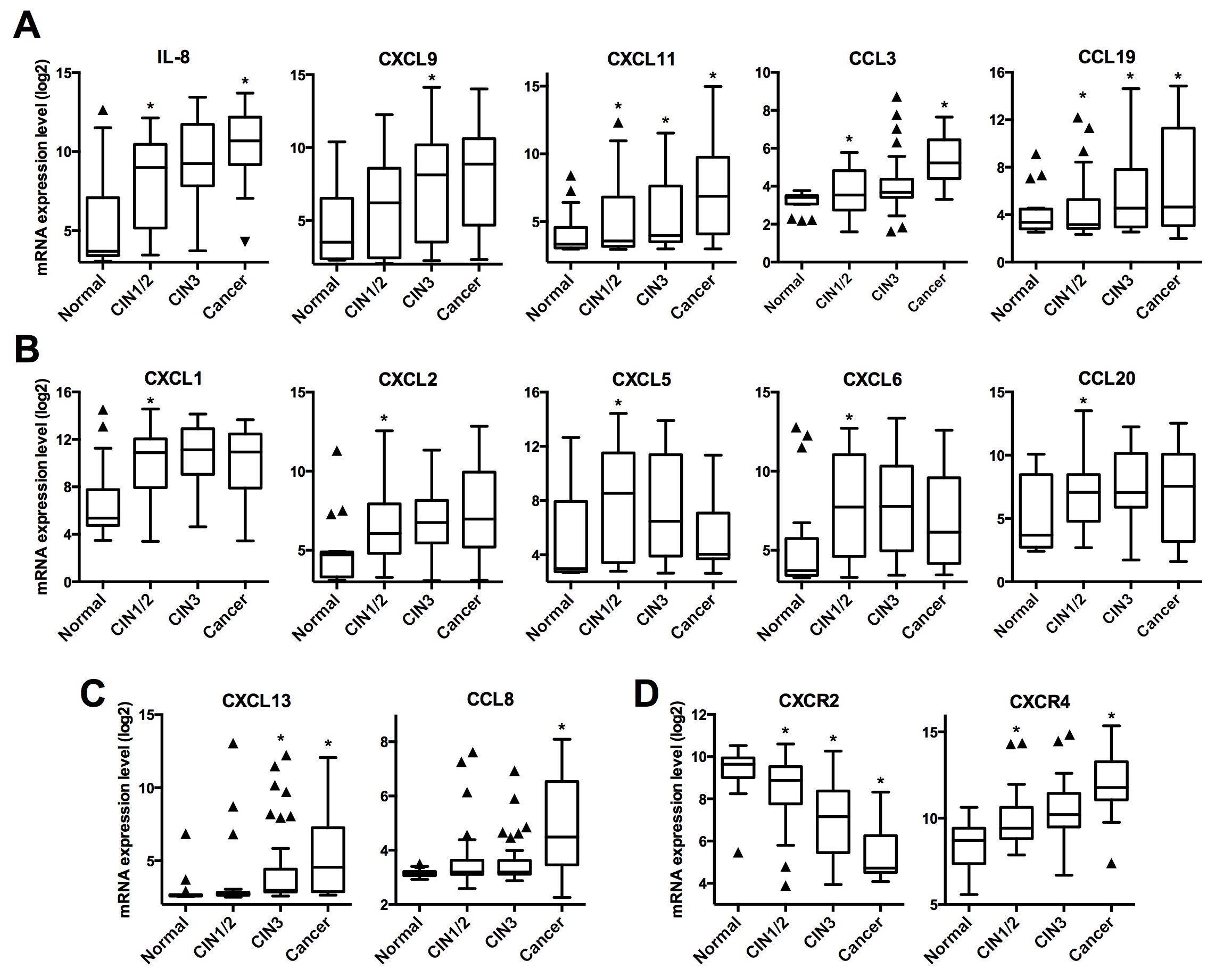

Supplement: Figure S1 — Chemokine expression is deregulated in HPV-associated cancer progression. Chemokines and chemokine receptors with significant changes of expression in CxCa progression are shown in the different panels. (A) IL-8, CXCL9, CXCL11, CCL3, and CCL19; (B) CXCL1, CXCL2, CXCL5, CXCL6, and CCL20; (C) CXCL13 and CCL8; and (D) CXCR2 and CXCR4. The gene expression data were analyzed from a global gene expression study of 128 cervical tissue samples in different disease stages: normal (n = 24); low-grade lesion (n = 36); high-grade lesion (n = 40); and cancer (n = 28) (4). Normalized fluorescence intensities (log2) of gene expression from each group are shown in box-and-whisker plots with Tukey’s method for outliers (black triangles) noted as distinct data points. P values were calculated between each transition (normal to CIN1/2, CIN1/2 to CIN3, and CIN3 to cancer) by Student’s t test. *, P < 0.05. Download [file mbo002162801sf1.jpg]

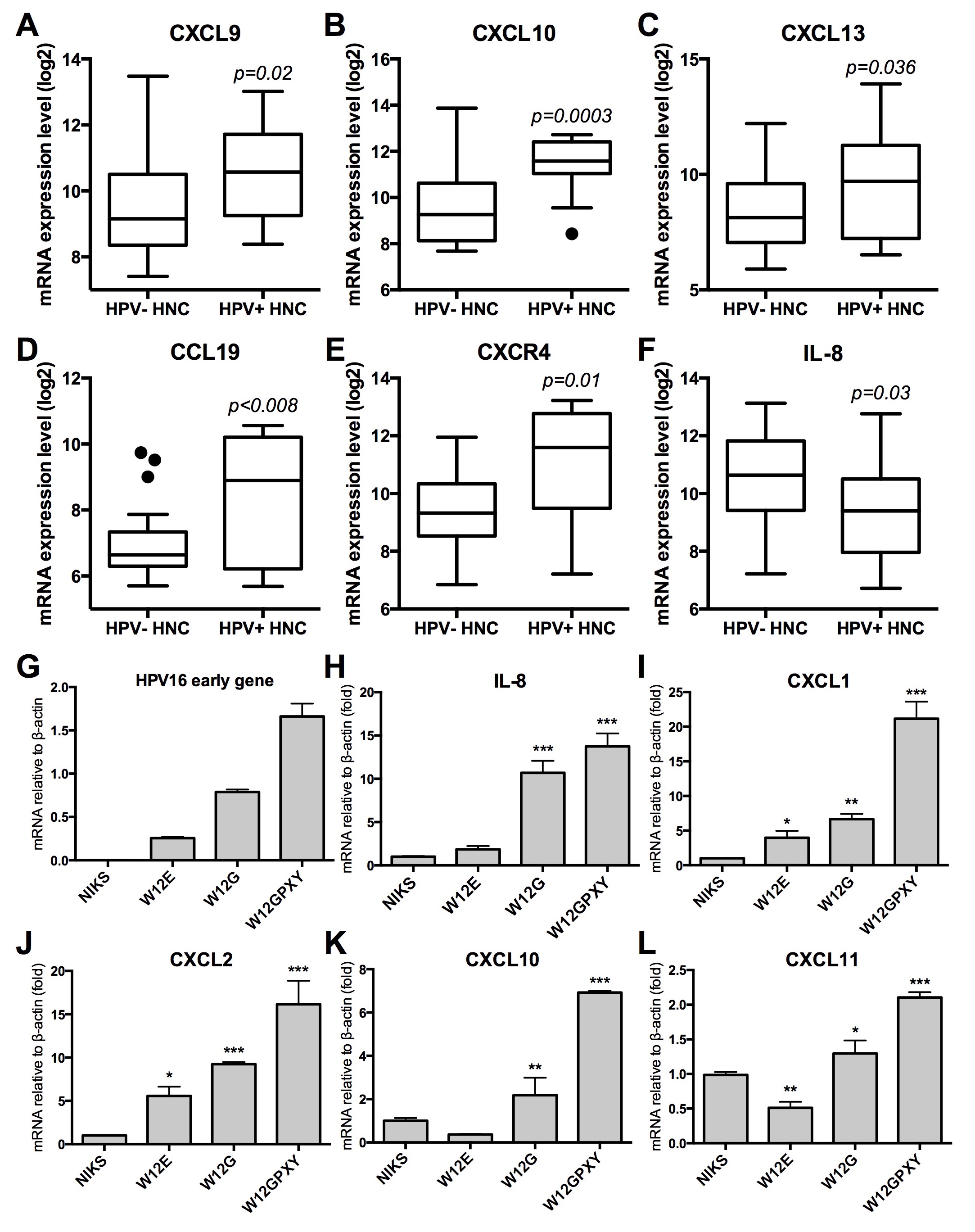

Supplement: Figure S2 — Proinflammatory chemokines are upregulated in HPV-positive HNCs and keratinocytes. (A to F) Gene expression levels of chemokines and chemokine receptors were analyzed with HPV-positive (n = 16) and HPV-negative (n = 26) HNCs from our previous global gene expression study (5) as described in the legends to Fig. 1 and Fig. S1 in the supplemental material. Normalized fluorescence intensities (log2) of gene expression from each group are shown in box-and-whisker plots with Tukey’s method for outliers (black circle) noted as distinct data points. The P values shown on each panel were calculated for HPV-negative and HPV-positive HNCs by Student’s t test. (G to L) Total RNA was extracted from NIKS, W12E, W12G, and W12GPXY keratinocyte lines. (G) HPV16 early gene transcript E1^E4 was measured by RT-qPCR, as previously described (59). (H to L) mRNA expression of IL-8, CXCL1, CXCL2, CXCL10, and CXCL11 were measured by RT-qPCR using specific primers (see Table S2 in the supplemental material), and normalized by β-actin mRNA. Data are shown as fold changes (± SD) to the mRNA level in NIKS cells. P values were determined by Student’s t test. *, P < 0.05; **, P < 0.001; ***, P < 0.0001. Download [file mbo002162801sf2.jpg]

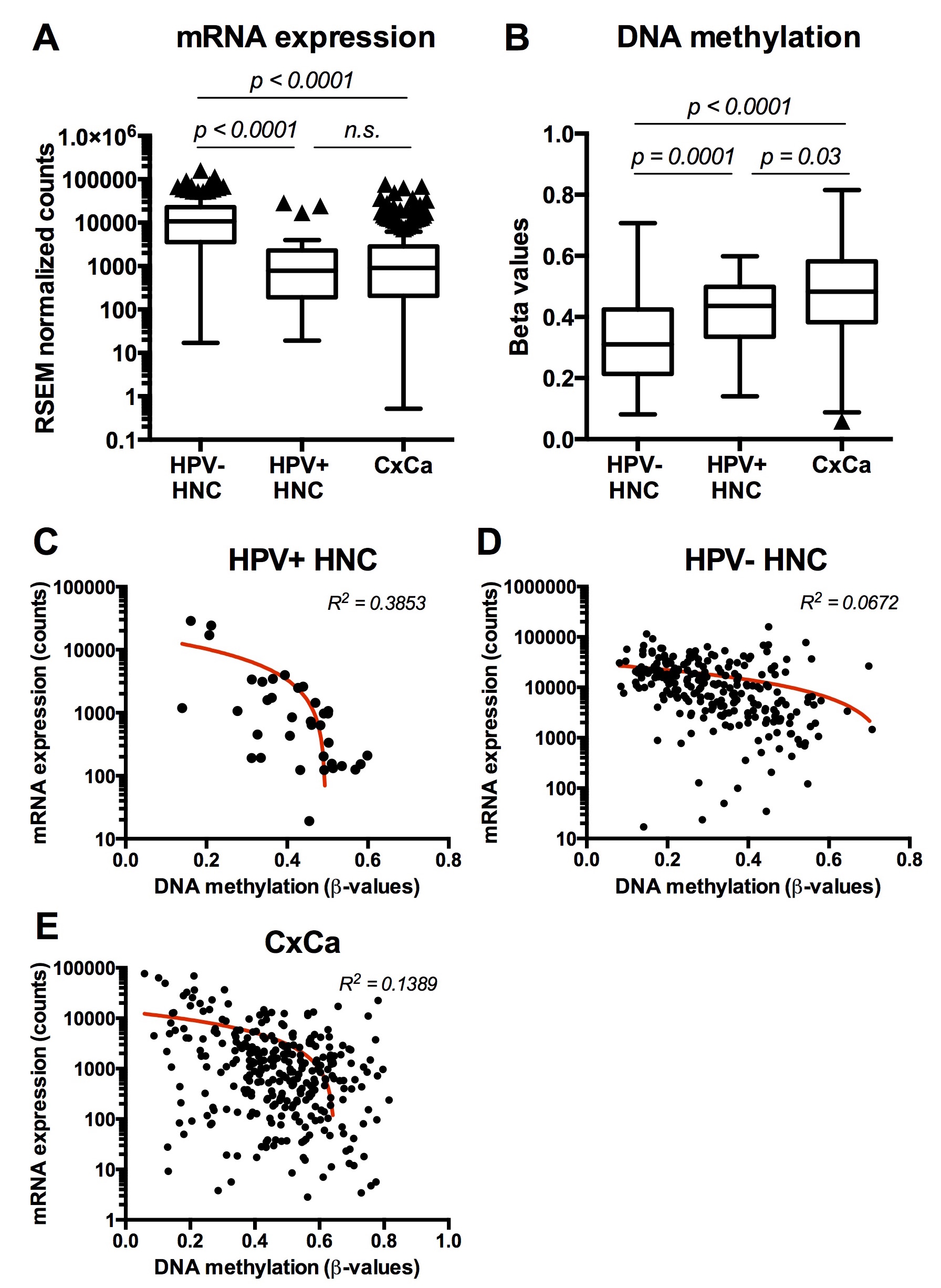

Supplement: Figure S3 — CXCL14 downregulation correlates with increased CXCL14 promoter methylation in HPV-positive HNC and CxCa. The TCGA data sets of CXCL14 RNA-seq RSEM (RNA-seq by expectation maximization) counts (mRNA expression) and beta values (DNA methylation) were obtained from cBioPortal (cbioportal.org): HPV-negative HNC, n = 243; HPV-positive HNC, n = 36 (23); CxCa, n = 309 (NCI, TCGA, Provisional). Normalized RSEM counts (A) and beta values (B) are shown in box-and-whisker plots with Tukey’s method for outliers (black triangles) noted as distinct data points. P values were determined by Student’s t test. n.s., not significant. Correlations between CXCL14 mRNA expression and DNA methylation were analyzed within HPV-positive (HPV+) HNC (C), HPV-negative (HPV−) HNC (D), and CxCa (E). The correlation coefficient (R2) was determined by linear regression using Prism software. Download [file mbo002162801sf3.jpg]

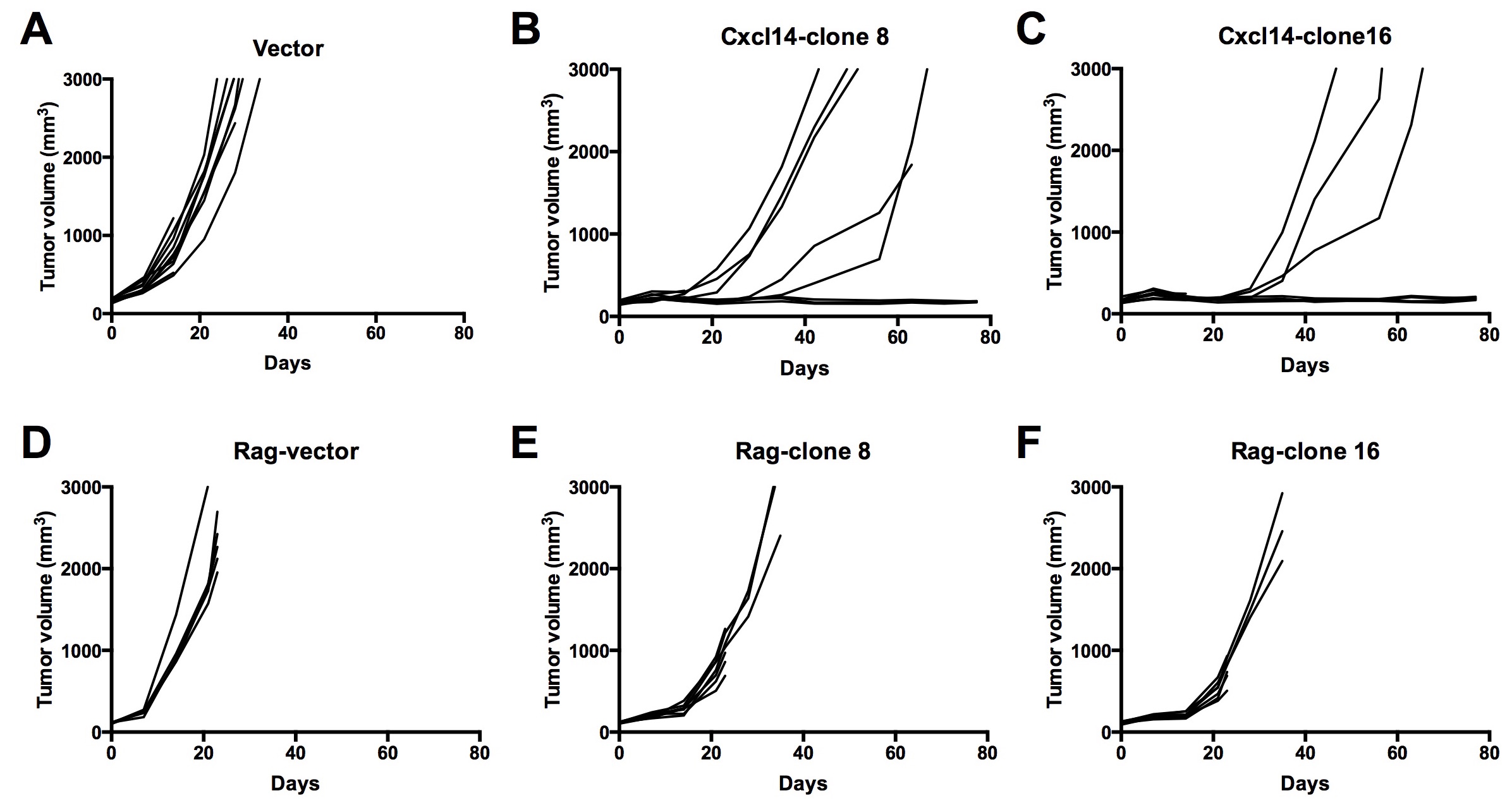

Supplement: Figure S4 — Restoration of Cxcl14 expression suppresses tumor growth in vivo. MOE/E6E7 cell clones reexpressing Cxcl14 (clones 8 and 16) and a vector containing MOE/E6E7 cell clone were injected into the rear right flank of wild-type C57BL/6 (A to C) and Rag1−/− (D to F) mice (n = 10 for each group of wild-type mice; n = 7 for each group of Rag1−/− mice). Tumor growth was determined every week by the following formula: volume = (width)2 × depth. Tumor growth curves of each mouse are shown. Download [file mbo002162801sf4.jpg]

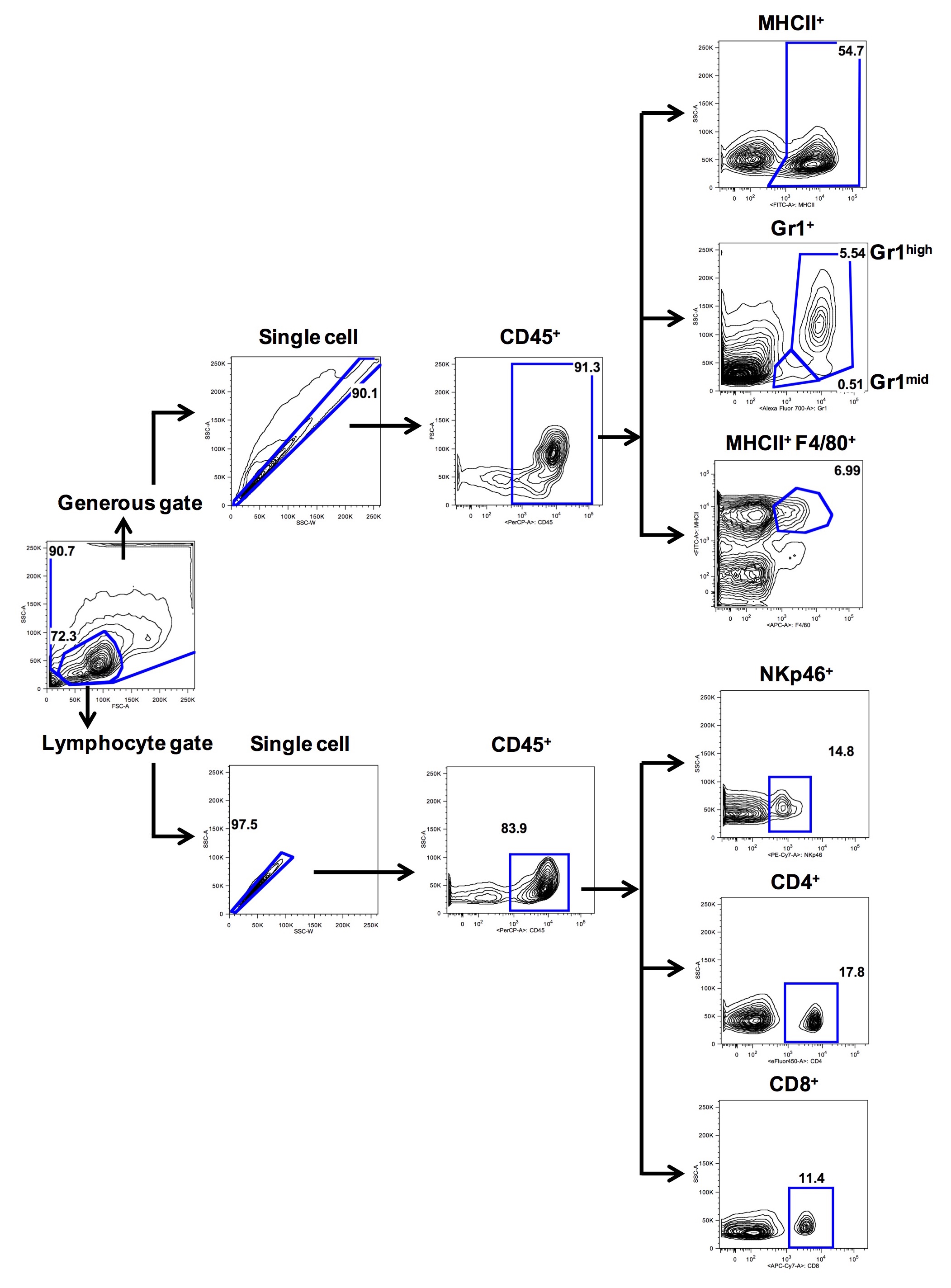

Supplement: Figure S5 — Gating strategy for flow cytometry. The whole spleen from a C57BL/6 mouse was homogenized and stained with a panel of antibodies conjugated to unique fluorophores. Single-stain and no-stain controls were used for fluorescence compensation. A generous large cell gate (forward scatter versus side scatter area), single cell gate (side scatter area versus side scatter width), and CD45+ gate (side scatter area versus CD45) were applied as parental gates before determining antigen-presenting cell (side scatter area versus MHCII), neutrophil (side scatter high, Gr1high), monocyte (side scatter low, Gr1mid), and macrophage (MHCII+ F4/80+) populations. A small cell lymphocyte gate (side scatter area versus forward scatter) and single cell gate (side scatter area versus side scatter width) were applied as parental gates to determine NK cell (CD45+ NKp46+), CD4+ T cell (CD45+ CD4+), and CD8+ T cell (CD45+ CD8+) populations. A representative example of the overall gating strategy is shown and was applied to TDLNs, distal lymph nodes, and spleens harvested from C57BL/6 mice injected with MOE/E6E7 cells with Cxcl14 or vector. Download [file mbo002162801sf5.jpg]

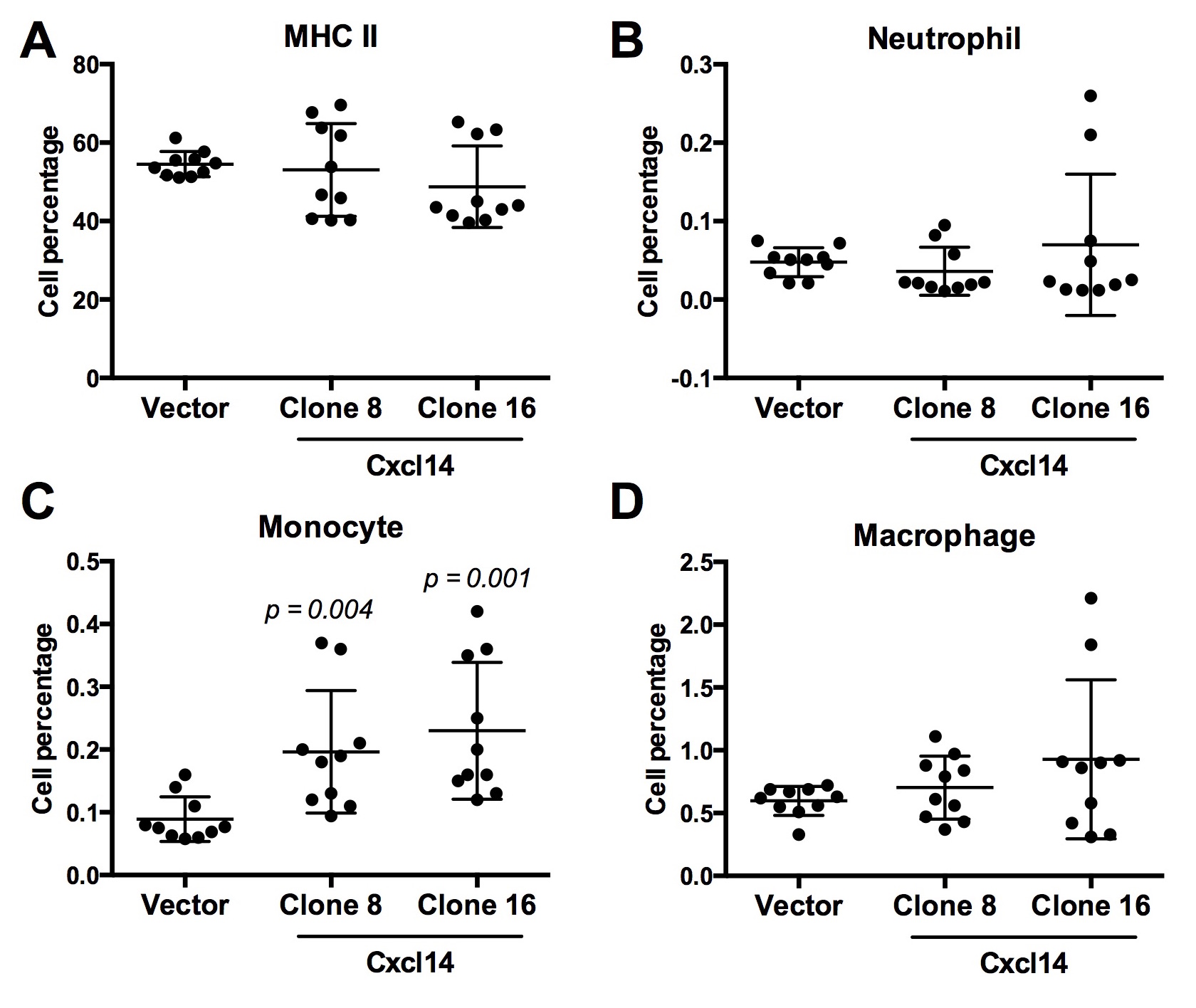

Supplement: Figure S6 — Changes of immune cell populations in TDLNs by Cxcl14 reexpression. MOE/E6E7 cells with Cxcl14 (clones 8 and 16) or the vector were injected into the right flank of C57BL/6 mice (n = 10 for each group). TDLNs were harvested 21 days postinjection. The percentages of antigen-presenting cell (A), neutrophil (B), monocyte (C), and macrophage (D) populations were determined by flow cytometry using specific antibodies as described in Materials and Methods. P values were determined between vector alone and clone 8 or clone 16 by Student’s t test. Download [file mbo002162801sf6.jpg]

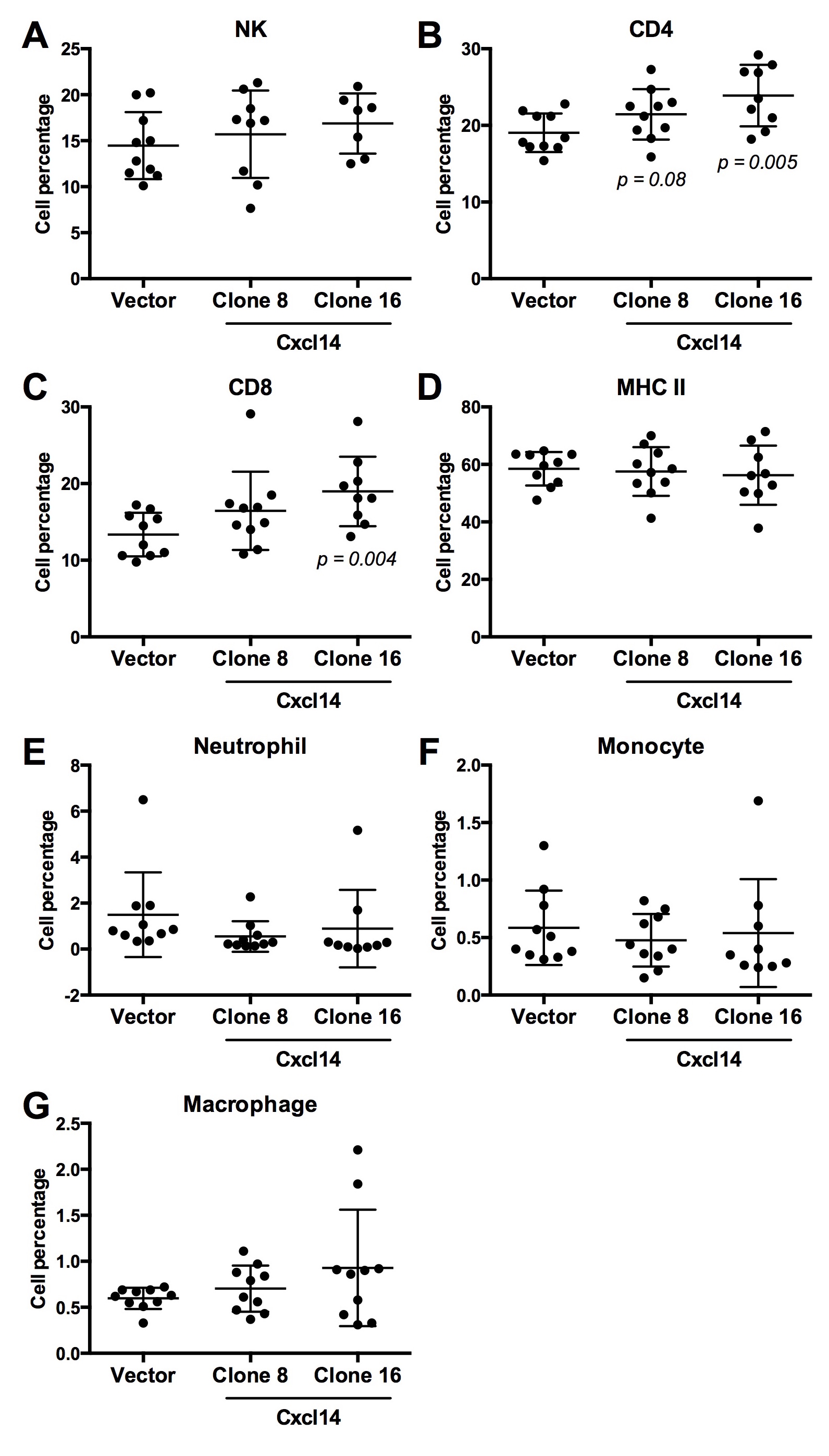

Supplement: Figure S7 — Changes of immune cell populations in spleens by Cxcl14 reexpression. MOE/E6E7 cells with Cxcl14 (clones 8 and 16) or the vector were injected into the right flank of C57BL/6 mice (n = 10 for each group). Spleens were harvested at 21 days postinjection. The percentages of NK cell (A), CD4+ T cell (B), CD8+ T cell (C), antigen-presenting cell (D), neutrophil (E), monocyte (F), and macrophage (G) populations were determined by flow cytometry using specific antibodies as described in Materials and Methods. P values were determined between vector alone and clone 8 or clone 16 by Student’s t test. Download [file mbo002162801sf7.jpg]
