# Supplementary material for: Suppression of Antitumor Immune Responses by Human Papillomavirus through Epigenetic Downregulation of CXCL14
Source: mBio. 2016 May 3;7(3):e00270-16. doi: 10.1128/mBio.00270-16 (PMC4959654; doi:10.1128/mBio.00270-16)
Supplement: Table S1 — Chemokine expression profiles of tissue specimens from cervix and head/neck. [file mbo002162801st1.pdf]

SUPPLEMENTAL TABLES

Table S1. Chemokine expression profiles of tissue specimens from cervix and head/neck.

Gene Symbol	Cervix					Head and neck		
	Expression level (Log2)				Fold	Expression level (Log2)		Fold
	Normal	Early	Late	Cancer	Cancer/normal	HPV+HNC	HPV-HNC	HPV+/-
**IL8**	5.277	7.316	9.255	10.482	**36.886**	9.521	10.543	**-2.031**
**CXCL9**	2.971	4.327	5.705	6.653	**12.835**	10.602	9.605	**1.995**
**CXCL1**	6.316	8.324	10.212	9.568	**9.527**	9.079	9.771	**-1.616**
**CXCR4**	7.941	8.732	9.082	10.729	**6.907**	10.839	9.422	**2.671**
**CXCL10**	6.407	7.482	8.19	9.154	**6.713**	11.427	9.699	**3.315**
**CXCL11**	3.365	4.144	4.214	5.975	**6.105**	7.326	6.426	**1.867**
**CXCL2**	3.216	4.169	5.215	5.673	**5.491**	7.375	7.157	**1.163**
**CCL19**	3.62	3.962	4.728	5.99	**5.169**	8.313	6.941	**2.587**
**CCL3**	3.175	3.502	4.234	5.425	**4.757**	8.386	8.190	**1.146**
**CXCL13**	2.31	2.411	3.353	4.361	**4.144**	9.586	8.328	**2.392**
**CCL20**	3.532	4.707	5.864	5.544	**4.033**	10.414	9.317	**2.140**
**CCL8**	3.011	3.498	3.5	4.978	**3.910**	7.194	7.245	**-1.036**
**CXCL6**	5.867	7.261	8.418	7.671	**3.492**	6.554	6.717	**-1.119**
**CCR1**	3.592	4.234	4.18	5.197	**3.042**	8.124	7.836	**1.221**
**CCR1**	4.588	5.004	5.525	5.986	**2.635**	8.046	7.734	**1.241**
**CCL11**	2.983	3.049	3.246	3.865	**1.843**	6.963	7.055	**-1.066**
**CXCL16**	7.82	8.214	8.803	8.667	**1.799**	9.653	9.710	**-1.040**
**CXCL3**	3.956	4.267	4.934	4.8	**1.795**	6.776	6.388	**1.308**
**CCL18**	3.413	3.391	3.6	4.202	**1.728**	9.000	8.333	**1.587**
**CCR7**	4.276	4.264	4.541	4.885	**1.525**	8.207	7.579	**1.545**
**CCL4**	6.184	6.469	6.48	6.772	**1.503**	9.167	8.856	**1.240**
**CCR2**	2.057	2.26	2.431	2.484	**1.344**	7.292	6.876	**1.334**
**CCL22**	2.504	2.62	3.206	2.724	**1.165**	8.778	8.698	**1.057**
**CCR10**	2.964	2.941	2.919	3.181	**1.162**	6.059	6.118	**-1.042**
**CCL13**	2.82	2.82	2.628	3.013	**1.143**	6.994	7.143	**-1.109**
**CCR6**	3.252	3.384	3.245	3.388	**1.099**	5.384	5.101	**1.217**
**CCL21**	2.828	2.515	2.761	2.961	**1.097**	6.853	6.867	**-1.010**
**CCL28**	6.634	6.705	6.943	6.741	**1.077**	7.873	7.876	**-1.002**
**CCR5**	5.722	5.95	5.932	5.798	**1.054**	10.640	10.195	**1.361**
**CCR9**	1.423	1.422	1.416	1.411	**-1.008**	6.599	6.600	**-1.001**
**CCR4**	2.2	2.214	2.197	2.187	**-1.009**	7.466	7.499	**-1.023**
**CCR2**	1.773	1.773	1.765	1.756	**-1.012**	6.284	6.451	**-1.123**
**CCL23**	3.68	3.678	3.59	3.661	**-1.013**	4.146	4.185	**-1.028**
**CCL17**	3.169	3.201	3.281	3.145	**-1.017**	6.019	6.024	**-1.004**
**CCR3**	1.786	1.79	1.77	1.76	**-1.018**	6.534	6.672	**-1.100**
**CCR8**	2.332	2.326	2.311	2.303	**-1.020**	7.761	7.846	**-1.060**
**CCL25**	2.414	2.415	2.397	2.384	**-1.021**	7.088	7.329	**-1.182**
**CCL24**	2.367	2.365	2.349	2.333	**-1.024**	6.073	6.145	**-1.051**
**CXCR5**	2.171	2.167	2.151	2.135	**-1.025**	6.562	6.565	**-1.002**
**CCL7**	3.562	3.603	3.551	3.524	**-1.027**	5.387	5.523	**-1.099**
**CCL27**	3.6	3.609	3.582	3.557	**-1.030**	6.446	6.396	**1.035**
**CXCR1 (IL8RA)**	2.285	2.268	2.261	2.241	**-1.031**	8.648	8.745	**-1.069**
**CCL1**	2.607	2.608	2.588	2.558	**-1.035**	6.016	6.195	**-1.133**
**CCL16**	2.179	2.173	2.093	2.091	**-1.063**	7.074	7.237	**-1.120**
**CXCR6**	4.513	4.674	4.737	4.4	**-1.081**	7.704	6.984	**1.647**
**CCL14/CCL15**	3.9	3.359	3.251	3.784	**-1.084**	7.643	7.769	**-1.091**
**CCL2**	8.624	9.347	8.719	8.458	**-1.122**	8.311	7.745	**1.480**
**CXCR3**	4.883	4.927	4.892	4.631	**-1.191**	8.115	7.946	**1.125**
**CX3CL1**	6.316	6.198	7.436	5.911	**-1.324**	8.693	8.304	**1.309**
**CCL5**	7.534	8.052	8.067	7.125	**-1.328**	9.037	8.180	**1.810**
**CXCL5**	4.254	5.357	5.732	3.658	**-1.512**	5.474	6.021	**-1.462**
**CCL26**	4.713	4.142	4.165	4.018	**-1.619**	7.047	7.344	**-1.229**
**CXCL12**	7.871	6.837	6.372	7.091	**-1.717**	7.854	7.259	**1.511**
**CXCR7**	10.698	11.061	11.132	9.902	**-1.736**	10.199	10.441	**-1.183**
**CXCL17**	11.107	11.597	11.911	9.98	**-2.184**	9.011	9.549	**-1.452**
**CX3CR1**	6.686	6.405	5.776	4.903	**-3.441**	7.243	6.901	**1.267**
**CXCR2 (IL8RB)**	9.508	9.192	7.523	5.86	**-12.536**	6.435	6.417	**1.012**
**CXCL14**	12.001	11.062	9.811	7.594	**-21.215**	9.271	10.949	**-3.200**
