# Supplementary material for: Suppression of Antitumor Immune Responses by Human Papillomavirus through Epigenetic Downregulation of CXCL14
Source: mBio. 2016 May 3;7(3):e00270-16. doi: 10.1128/mBio.00270-16 (PMC4959654; doi:10.1128/mBio.00270-16)
Supplement: Table S2 — Quantitative PCR primers. [file mbo002162801st2.pdf]

Table S2. Quantitative PCR primers.

Gene	Direction	Sequences
**HPV16 E1^E4**	Forward	5'-AAATGACAGCTCAGAGGAGGAG-3'
	Reverse	5'-GAGTCACACTTGCAACAAAAGG-3'
**β-Actin**	Forward	5'-TCACCCACACTGTGCCCATCTA-3'
	Reverse	5'-TGAGGTAGTCAGTCAGGTCCCG-3'
**CXCL14**	Forward	5'-GGAAATGAAGCCAAAGTACCC-3'
	Reverse	5'-AGGCGTTGTACCACTTGATGA-3'
**IL-8**	Forward	5'-AAGAAACCACCGGAAGGAAC-3'
	Reverse	5'-AGCACTCCTTGGCAAAACTG-3'
**CXCL1**	Forward	5'-CTTCCTCCTCCCTTCTGGTC-3'
	Reverse	5'-GAAAGCTTGCCTCAATCCTG-3'
**CXCL2**	Forward	5'-GCTTCCTCCTTCCTTCTGGT-3'
	Reverse	5'-GGGCAGAAAGCTTGTCTCAA-3'
**CXCL10**	Forward	5'-AGGAACCTCCAGTCTCAGCA-3'
	Reverse	5'-CAAAATTGGCTTGCAGGAAT-3'
**CXCL11**	Forward	5'-AGTGTGAAGGGCATGGCTAT-3'
	Reverse	5'-GCCTTGCTTGCTTCGATTTG-3'
**DNMT1**	Forward	5'- TTCTGTTAAGCTGTCTCTTTCCA-3'
	Reverse	5'-TGCTGAAGCCTCCGAGAT-3'
